# Transcriptomic Evidence of the Immune Response Activation in Individuals With Limb Girdle Muscular Dystrophy Dominant 2 (LGMDD2) Contributes to Resistance to HIV-1 Infection

**DOI:** 10.3389/fcell.2022.839813

**Published:** 2022-05-13

**Authors:** Francisco Diez-Fuertes, María Rosa López-Huertas, Javier García-Pérez, Esther Calonge, Mercedes Bermejo, Elena Mateos, Pilar Martí, Nuria Muelas, Juan Jesús Vílchez, Mayte Coiras, José Alcamí, Sara Rodríguez-Mora

**Affiliations:** ^1^ AIDS Immunopathogenesis Unit, National Center of Microbiology, Instituto de Salud Carlos III, Madrid, Spain; ^2^ Centro de Investigación Biomédica en Red de Enfermedades Infecciosas (CIBERINFEC), Madrid, Spain; ^3^ Neuromuscular Diseases Unit, Neurology Department, Hospital Universitari i Politècnic La Fe, Valencia, Spain; ^4^ Centro de Investigación Biomédica en Red de Enfermedades Raras (CIBERER), Valencia, Spain; ^5^ Infectious Diseases Unit, IDIBAPS, Hospital Clinic, University of Barcelona, Barcelona, Spain

**Keywords:** HIV-1, LGMDD2, transcriptome, chemokines, inflammation pathway

## Abstract

LGMDD2 is a rare form of muscular dystrophy characterized by one of the three heterozygous deletions described within the *TNPO3* gene that result in the addition of a 15-amino acid tail in the C-terminus.TNPO3 is involved in the nuclear import of splicing factors and acts as a host cofactor for HIV-1 infection by mechanisms not yet deciphered. Further characterization of the crosstalk between HIV-1 infection and LGMDD2 disease may contribute to a better understanding of both the cellular alterations occurring in LGMDD2 patients and the role of TNPO3 in the HIV-1 cycle. To this regard, transcriptome profiling of PBMCs from LGMDD2 patients carrying the deletion c.2771delA in the *TNPO3* gene was compared to healthy controls. A total of 545 differentially expressed genes were detected between LGMDD2 patients and healthy controls, with a high representation of G protein-coupled receptor binding chemokines and metallopeptidases among the most upregulated genes in LGMDD2 patients. Plasma levels of IFN-β and IFN-γ were 4.7- and 2.7-fold higher in LGMDD2 patients, respectively. An increase of 2.3-fold in the expression of the interferon-stimulated gene MxA was observed in activated PBMCs from LGMDD2 patients after *ex vivo* HIV-1 pseudovirus infection. Thus, the analysis suggests a pro-inflammatory state in LGMDD2 patients also described for other muscular dystrophies, that is characterized by the alteration of IL-17 signaling pathway and the consequent increase of metallopeptidases activity and TNF response. In summary, the increase in interferons and inflammatory mediators suggests an antiviral environment and resistance to HIV-1 infection but that could also impair muscular function in LGMDD2 patients, worsening disease evolution. Biomarkers of disease progression and therapeutic strategies based on these genes and mechanisms should be further investigated for this type of muscular dystrophy.

## Introduction

Limb-Girdle Muscular Dystrophy (LGMD) is a heterogeneous group of genetic disorders characterized by a progressive and predominantly proximal muscle weakness affecting the pelvic and shoulder girdles with histological signs of muscle degeneration and regeneration (Bushby, 2009). More than 30 subtypes of LGMD have been described, including 5 autosomal dominant and 26 autosomal recessive based on the inheritance pattern. LGMDD2 is a rare autosomal dominant subtype of LGMD that was firstly reported in 2001 in a large Spanish family, affecting currently 32 individuals (Gamez et al., 2001). LGMDD2 was initially associated to the heterozygous deletion c.2771delA in the position 128,957,256 of the chromosome 7 (rs587777430), within the *TNPO3* gene and affecting the adenine of the TAG stop codon in the exon 21. More recently, other mutations in the same exon have been associated to LGMDD2, including c.2767delC (rs1563083759) observed in two individuals of the same Hungarian family (Pál et al., 2019) and c.2757delC found in three members of a Swedish family (Vihola et al., 2019). All these three frameshift variants in exon 21 of TNPO3 generate a transcript encoding 938 amino acids isoform of TNPO3 with fifteen additional residues in the C-terminus compared with the wild type protein of 923 amino acids. Another LGMD sporadic case found in a single individual shares the clinical phenotype described for LGMDD2 with adult onset disease, characterized by a slow progression of pelvic and shoulder girdle weakness (Gibertini et al., 2018). However, this individual case differs in some specific indicators of LGMDD2 (such as dysphagia, arachnodactyly, and dysarthria), and was associated with the heterozygous c.G2453A (R818P) mutation in exon 20 of TNPO3 (rs587777431) (Gibertini et al., 2018). Both TNPO3-wildtype and TNPO3-mutated proteins are translated in these patients (Rodríguez-Mora et al., 2019).

TNPO3 is a member of the importin beta/transportin family involved in the nuclear import of the essential splicing factors termed proteins serine/arginine (SR)-rich proteins as SF2, SC35 and cleavage and polyadenylation specificity factor subunit 6 (CPSF6) (Kataoka et al., 1999; Lai et al., 2001; Maertens et al., 2014). TNPO3 associates with target cargo proteins in the cytoplasm through the C-terminal domain and therefore importin function is altered in the mutated protein (Melià et al., 2013; Torella et al., 2013; Rodríguez-Mora et al., 2019).

Besides its role in LGMDD2 disease, TNPO3 was identified as a HIV-1 co-factor in three independent genome-wide siRNA screens (Brass et al., 2008; König et al., 2008; Zhou et al., 2008; Bushman et al., 2009) although the precise mechanism of its antiviral action has been a matter of controversy (Hilditch and Towers, 2014). Mechanistically, TNPO3 regulates the nuclear transport of CPSF6, which is a critical co-factor of HIV-1 capsid stability and contributes to the ability of HIV-1 to evade immune sensors (De Iaco et al., 2013; Fricke et al., 2013; Rasaiyaah et al., 2013).

HIV-1 viral cycle includes virion RNA retrotranscription into cDNA and its integration within the DNA genome of the infected cell, establishing the long-term viral latency that prevents disease cure. HIV-1 cDNA gains access to the nucleus using the cellular nuclear transport machinery located at the nuclear pore, in the form of a pre-integration complex (PIC), consisting of viral cDNA as well as some HIV-1 proteins and host cell proteins (Piller et al., 2005). Some studies have shown that TNPO3 participates in the nuclear import of PICs whereas others suggested that TNPO3 can play an indirect role in viral integration through its interaction with CPSF6 or LEDGF/p75, which bind the capsid and integrase HIV-1 proteins, respectively (Krishnan et al., 2010; Larue et al., 2012; Hilditch and Towers, 2014; Tsirkone et al., 2017).

Because TNPO3 is a common factor in both HIV-1 infection and LGMDD2 disease progression, our group has previously studied the susceptibility to *in vitro* HIV-1 infection in T cells isolated from LGMDD2 patients. In 32 individuals of the same Spanish/Italian family affected by LGMDD2, we demonstrated that peripheral blood mononuclear cells (PBMCs) from these patients are resistant to HIV-1 infection *in vitro* (Rodríguez-Mora et al., 2019). The mechanism behind this resistance to HIV-1 infection was due to a strong decrease in HIV-1 integration probably related to the defective transport of CPSF6 by TNPO3 and the formation of TNPO3-CPSF6-capsid complexes. An alternative or complementary hypothesis to explain the resistance to HIV-1 infection of CD4^+^ T lymphocytes of LGMDD2 patients proposes that capsid retention in the cytosol due to decreased nuclear import can provoke increased levels of retro-transcribed DNA in the cytosol. These complexes would be sensed and trigger class I interferon (IFN) and inflammatory mechanisms leading to increased antiviral activity and inhibition of HIV-1 infection (Hilditch and Towers, 2014).

Despite the fact that mutation in TNPO3 is the origin and cause of LGMDD2, the mechanisms of muscle damage are not fully understood. It should be noted that LGMDD2 patients show a wide variability in clinical symptoms, severity of disease and age onset. Accordingly, myopathologic profiles are different among patients but autophagosomes and inflammatory phenomena have been described in muscle biopsies (Cenacchi et al., 2013; Peterle et al., 2013; Torella et al., 2013) pointing to autophagy and inflammation as potential mechanisms of damage. Muscle inflammation is common in other muscular dystrophies (Georganopoulou et al., 2021; González-Mera et al., 2021), and some non-disease specific treatments with a therapeutic effect that reduces inflammation have been tested in patients with other muscular dystrophies and LGMD subtypes (Georganopoulou et al., 2021).

Therefore, further studies to describe the crosstalk between HIV-1 infection and LGMDD2 may be important for a better understanding of cellular alterations occurring in subjects living with LGMDD2 that contribute to both muscle disease and resistance to HIV-1 infection. To this regard, a high-throughput transcriptome profiling of peripheral blood mononuclear cells from LGMDD2 patients compared to healthy controls has been performed. Special interest was focused on pathways related to immune response and inflammation that play a role in both diseases.

## Materials and Methods

### LGMDD2 Patients and Healthy Controls

Ten LGMDD2 patients and ten healthy controls were recruited for this study. All LGMDD2 patients had been regularly submitted to a close clinical follow-up in University Hospital La Fe, Valencia. Healthy controls were matched in age and genders and were recruited within not affected by the disease, which was previously determined by DNA sequencing of *TNPO3* gene.

### PBMCs Isolation

PBMCs from LGMDD2 patients and healthy controls were isolated by centrifugation using Ficoll-Hypaque gradient (GE Healthcare). Plasma was aliquoted and stored at -80°C until analysis. PBMCs were cultured in RPMI 1640 medium (Biowhitaker, Walkersville, MD) supplemented with 10% fetal calf serum (FCS), 2 mM L-glutamine, 100 μg/ml streptomycin, 100 units/ml penicillin (Bio Whittaker, Walkersville, MD). PBMCs were then activated with purified antihuman CD3 (clone OKT3), CD28 (clone CD28.2) (Biosciences, San Diego, CA, United States) and 300 U/ml interleukin-2 (IL-2) (Chiron, Emeryville, CA) for 3 days.

### RNA Isolation

Total RNA from activated PBMCs was isolated from 5 × 10^6^ cells of each samples using RNeasy Mini Kit (Qiagen), according to the manufacturer’s instructions. The concentration and quality of all samples was analyzed with the kit RNA 6000 Nano in 2100 Bioanalyzer (Agilent Technologies) for valued the RNA Integrity Number (RIN) value. The samples with RIN values higher than 8.5 were used to create the libraries.

### Next Generation Sequencing

A total of 4 ug of RNA was used to generate a next-generating sequencing library using the ScriptSeq™ v2 RNA-Seq Library Preparation Kit (Illumina, San Diego, CA, United States), according to manufacturer’s instructions. Ribo-Zero rRNA Removal Kit (Illumina, San Diego, CA, United States) assisted to removal the ribosomal RNA, before purifying the samples with RNeasy Mini Kit (Qiagen). Subsequently, RNA was fragmented to produce an average RNA fragment size of 300 bp and cDNA was obtained using Star Script Reverse Transcriptase (Thermofisher). After tagging the 3′end of cDNA, the libraries were purified by the Agencourt AMPure XP System magnetic beads (Beckman Coulter). The tagged cDNA was barcoded by ligation with unique adaptor sequences to allow pooling of samples into groups of 10. Finally, DNA libraries were purified using the Agencourt AMPure XP System and the quality of the libraries was determined using the High Sensitivity DNA kit (Agilent Bioanalyzer). The libraries were sequenced using the NextSeq 500/550 high output kit v2.5 for 75 cycles (Illumina, San Diego, CA, United States) on Ilumina NextSeq 500 platform in the Genomics Unit of the National Center for Microbiology in the Instituto de Salud Carlos III (Madrid, Spain).

### Assessment of Sequence Quality and Trimming

Illumina sequences obtained from each sample were analyzed by fastQC tool (http://www.bioinformatics.babraham.ac.uk/projects/fastqc/) to evaluate sequence yield, base quality, GC profile, k-mer distribution, overrepresented sequences and primer contamination.

The Trimmomatic tool (http://www.usadellab.org/cms/?page=trimmomatic) is used to trim the adapter and/or primer sequences from the ends of the fastq generated. The software trims the end bases below a Phred quality score of 3 or any bases in a 4-base-wide sliding window when the average quality per base drops below 15. The program also clips adapters/primers from the sequences by allowing two seed mismatches. Any sequences trimmed to fragments shorter than 30 bases are removed from the output.

### Preparation of the Reference Genome

The trimmed sequencing reads are mapped to the transcripts derived from the human reference genome (GRCh38). The reference index files required by Bowtie2 mapping program and the transcript-specific reference sequences are generated from the reference fasta file and the corresponding annotation (gtf file) by the ‘rsem-prepare-reference’ command available in RSEM expression analysis software.

### Mapping and the Calculation of Expression Values

The ‘rsem-calculate-expression’ command from the RSEM expression analysis software is used to map single-end reads to the reference transcripts. The RNA expression values at gene and isoform levels are calculated using the expectation-maximization (EM) algorithm as implemented by the RSEM program. Multiple threads of eight or more are used to generate alignments mapped to genomic coordinates, while tagging reads with nonunique alignments (--tag), calculating 95% credibility intervals (--calc-ci) and posterior mean estimates (--calc-pme) and allowing insertions in the range of 1–500 bases (--fragment-length-min/max).

### Differential Expression

The number of fragments that are derived from a given isoform or gene using RSEM´s approximate maximum likelihood estimates was employed for differential expression between samples from LGMDD2 patients and healthy controls. An R package called EBSeq for differential expression analysis using RNA-Seq data was used. EBSeq is an empirical Bayesian approach used for the differential expression analysis based on the negative binomial distribution. Differentially expressed (DE) genes or isoforms with false discovery rate (FDR) controlled at 5% between LGMDD2 patients and healthy controls were the genes with a posterior probability of being differentially expressed (PPDE) greater than 0.95. The mRNA abundance of several DE genes was confirmed by quantitative reverse transcription-PCR (qRT-PCR). Relative abundance of each mRNA was quantified using SYBR Select Master mix (Life Technologies) and a set of gene specific primers (Supplementary Figure S1A) in a StepOne Real-Time PCR system (Applied Biosystems). The thermal cycling comprised a single step of 95°C for 10 min; and 40 cycles of 95°C for 15 s; and 60°C for 1 min. Gene expression was normalized against ACTB and the relative mRNA abundance in patients compared with controls was determined using the delta-delta of threshold cycle (2^−ΔΔCt^) as described earlier (Schmittgen et al., 2008).

### Functional Annotation

BioMart was used to convert gene symbols to unique Ensembl gene identifiers. DE gene set enrichment analysis was carried out using Kobas 3.0, calculating the probability of enrichment controlled at 5% against the following databases: Gene Ontology Slim, KEGG pathway and KEGG disease (Xie et al., 2011). DE GO terms were summarized by reducing redundant terms and visualized in semantic similarity-based plots using REVIGO and the tmPlot function of the R package treemapu (Supek et al., 2011). The presence of members of the human spliceosome among DE genes was assessed by the spliceosome database (Cvitkovic and Jurica, 2013) and ASpedia (Hyung et al., 2018).

### Determination of Biomarkers Concentrations in Plasma

The plasma of all participants, previously isolated and stored at -80°C, was used to measure the concentration of IFN-beta (IFN-β) and IFN-gamma (IFN-γ) Human Magnetic Luminex Screening Assays (LXSAHM; R&D Systems, Inc. Minneapolis, MN, United States), according to the manufacturer’s instructions. Duplicates measurements were performed per sample and a standard curve was created on each plate. Microparticle cocktail and biotin-antibody cocktail were added to each individual sample or standard and co-incubated on a shaker overnight (800 rpm, 4°C). Samples were washed and incubated with streptavidin-PE for 30 min (800 rpm, 20°C). After washing, the samples were resuspended in 100 μL wash buffer and analyzed with the software on Bio-Plex 200 detection platform (Bio-Rad, California, United States). The concentration of each analyte was measured in relation to the individual calibration curve and the results were expressed in pg/ml.

### HIV-1 Infection and Analysis of the Expression of MxA by Flow Cytometry

After activation of PBMCs from LGMDD2 patients and healthy controls with CD3/CD28/IL-2 for 3 days, 5 × 10^6^ cells were infected with 1 ng p24 of NL4.3-Renilla virus or VSV-pseudotyped ΔEnv-NL4.3-Luc virus per million of cells by spinoculation for 30 min at gently rotation at room temperature and then centrifuged at 1500 rpm for 30 min at 25°C. Plasmids pNL4.3-Renilla, pNL4.3-Luc-R_E, and pcDNA-VSV (express G protein of vesicular stomatitus virus) (Connor et al., 1995; He et al., 1995) were used to obtained the infectious supernatants from calcium phosphate transfection of HEK293T cells (provided by the existing collection of the Instituto de Salud Carlos III, Madrid, Spain). After washing with PBS 1X, infected cells were cultured for 5 days with IL-2. Cells were then collected, washed with PBS 1X, fixed with 1% paraformaldehyde (PFA), and permeabilized with 1% Tween-20. Cells were intracellularly stained with a monoclonal antibody for IFN-induced human myxovirus resistance protein 1 (MxA) and a secondary monoclonal antibody conjugated to PE (Becktn-Dickinson). Cells were analyzed using FACS Calibur cytometer (Becton Dickinson Biosciences) and Flowing software (Turku Bioscience, Turku, Finland).

### Statistical Analysis

Statistical analysis was performed using GraphPad Prism 8.0 Software (GraphPad Software Inc., San Diego, CA, United States). Shapiro-Wilk´s method was used as normality test. Statistical significance comparing IFN levels in plasma and IFN-induced production of MxA between LGMDD2 patients and healthy controls was calculated using unpaired, nonparametric Mann-Whitney *t*-test. Differences were considered statistically significant when *p* < 0.05. Differentially expressed genes between LGMDD2 patients and healthy controls were estimated as described above.

## Results

### Clinical Characteristics

Ten LGMDD2 patients were recruited for this study of a unique Spanish family of which two patients belonged to the third generation, four patients to the fourth generation and four patients to the fifth generation. Most of them were females (60%) with median age at onset of 10.88 years. The most common symptoms of LGMDD2 were proximal weakness at pelvic-femoral (80%), scapular-humeral (50%), distal weakness at leg (70%) and hand atrophy (50%). Most of them (60%) reached stage 4 of the Vignos scale with median age at 27.83 years and with median Brooke scale at 2.67. The serum creatine kinase (CK) was measured in half of the LGMDD2 patients with a median value at 382.25 (Table 1).

**TABLE 1:** Clinical characteristics of LGMDD2 patients who were recruited for this study.

ID	Gender (male/female)	Age at onset	Proximal weakness/atrophy	Distal weakness/atrophy	Brooke scale	Vignos scale	Age stage 4. Vignos scale	Serum CK
III.1	Female	−	−	−	1	0	−	−
III.12	Male	40	SH, PF	HAW/LW	3	6	48	350
IV.1	Male	−	−	−	−	0	−	−
IV.26	Male	5	SH, PF	HA/LW	4	7	20	295
IV.29	Female	7	SH, PF	HA/LW	2	3	-	NA
IV.31	Female	18	SH, PF	HAW/LW	2	4	31	744
V.1	Male	12	PF	−/LW	4	4	25	−
V.2	Female	3	PF	HA/LWA	4	4	18	−
V.30	Female	1	SH, PF	HA/LW	3	4	25	NA
V.36	Female	1	PF	HA/LW	1	3	−	140

SH, scapular-humeral; PF, pelvic-femoral; HAW, hand atrophy and weakness; LW, leg weakness; HA, hand atrophy; CK, creatine kinase; NA, not avaible.

### Overall Characteristics of Mapping

The sequencing reads of the 20 samples were split into three groups: unmapped, uniquely-mapped (if maps only to a single gene), and “multireads” (if maps to multiple genes). A mean of 34,846,774 reads per sample was obtained, reaching a mean alignment rate of 67.8% (55.0% multireads plus 12.8% of uniquely-mapped reads). The alignment rate of the libraries varied between 56.1 and 76.6%.

### Differentially Expressed Genes

**FIGURE 1:**
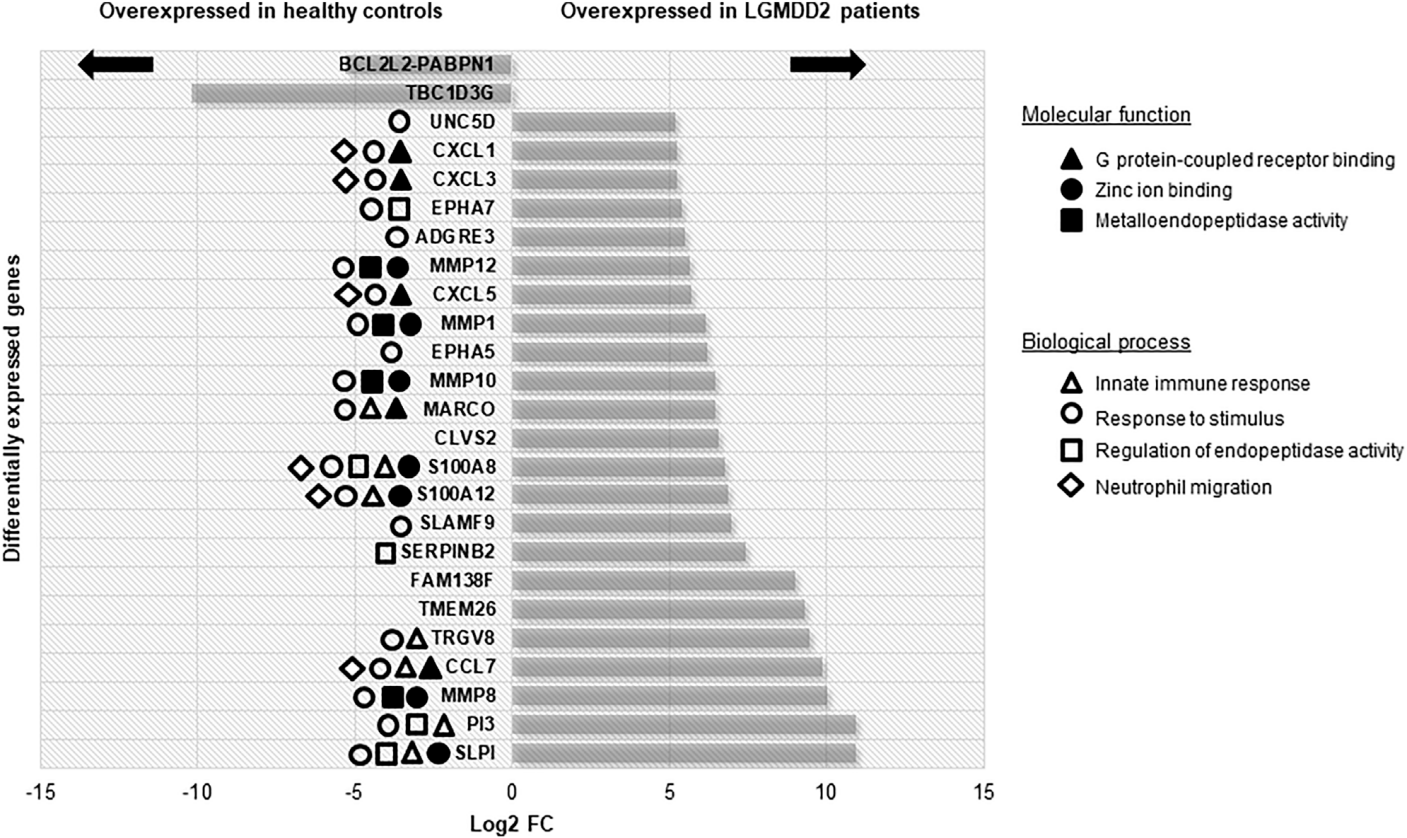
Differentially expressed genes with higher FC between LGMDD2 patients and healthy controls. Functional annotation of these genes is shown according to the legend.

A total of 535 DE genes were identified in the comparison between LGMDD2 patients and healthy controls (Supplementary Table S1). Among the top 25 DE genes, we found an upregulation in LGMDD2 patients of several genes implicated in G protein-coupled receptor binding chemokines (CXCL1, CXCL3, CXCL5, and CCL7), zinc ion binding proteins (MMP1, MMP8, MMP10, MMP12, S100A8, and S100A12), and metallopeptidase activity (MMP1, MMP8, MMP10, and MMP12). These molecular functions are mainly related to innate immunity and the response to inflammatory stimuli (Figure 1). Three long non-coding RNAs (lncRNAs) were also among the most differentially expressed transcripts between LGMDD2 patients and healthy controls, including RP11-439A17.9, RP11-483E23.2 and AC113404.1, which are located nearby FCGR1B (Fc fragment of IgG receptor Ib), NDVFS1 (NADH:ubiquinone oxidoreductase core subunit S1), and SVC2 (synaptic vesicle glycoprotein 2C). We also found differences in the small Cajal body-specific RNA 2 (SCARNA2). Higher relative abundance of several of these mRNAs were confirmed by qRT-PCR, including the metallopeptidases MMP1, MMP10, MMP12, the chemokines CXCL1, CXCL3, and CXCL5 and other genes involved in inflammatory processes such as PI3 and S100A8 (Supplementary Figure S1B).

**FIGURE 2:**
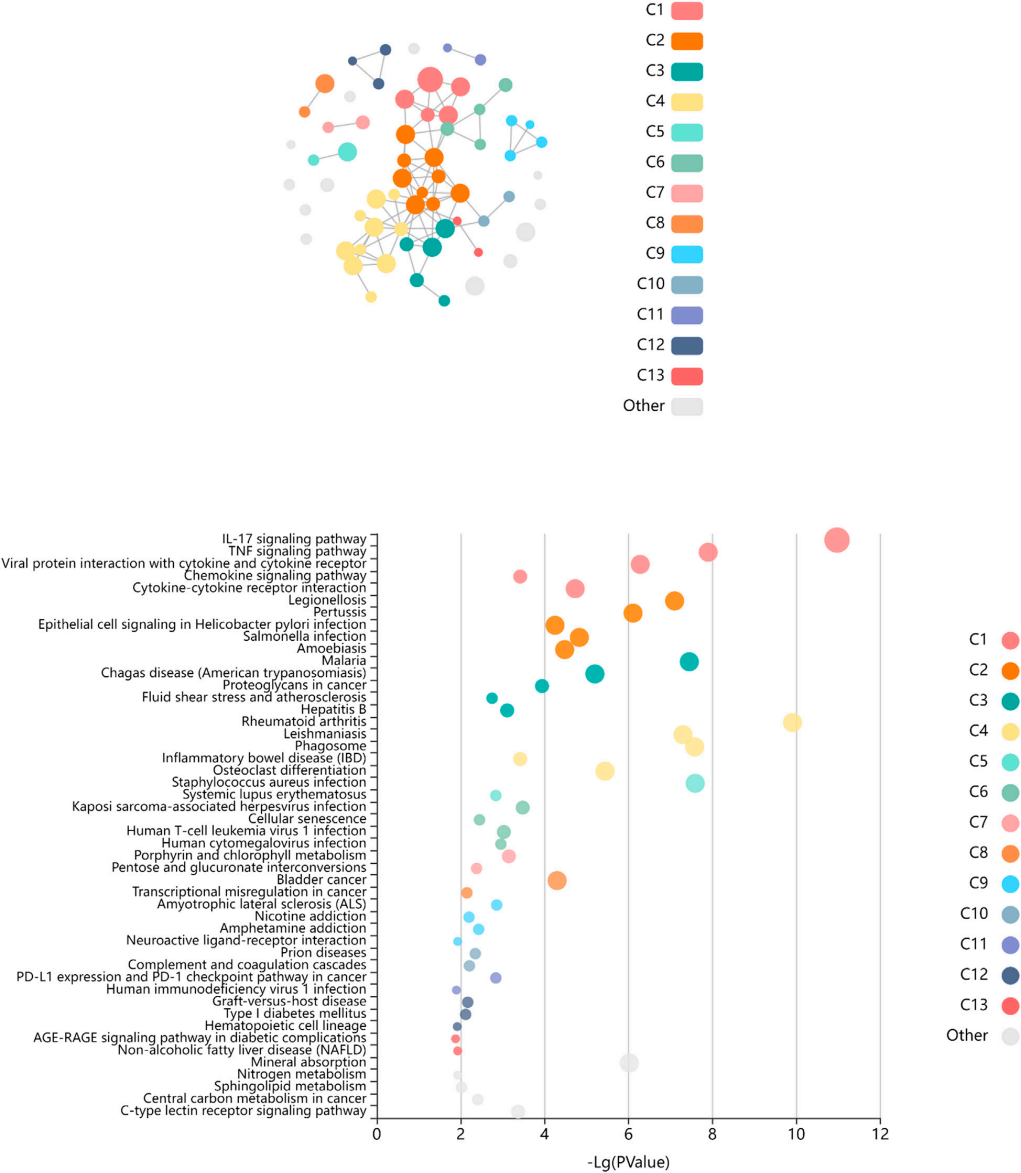
Enriched pathways of KEGG database associated to differentially expressed genes between LGMDD2 patients and healthy controls. Each node of the network represents an enriched term, the node color represents different clusters, and the node size represent 5 levels of FDR-corrected p-values (*p* < 0.05, *p* < 0.01, *p* < 0.001, *p* < 0.0001 and *p* < 0.00001). The FDR-corrected p-value associated to each specific KEGG pathway is showed in the bubble plot.

Among the genes overexpressed in healthy controls, pseudogenes and non-coding RNAs were the ones with higher fold change (FC) values. A lower expression of the following two genes was found in LGMDD2 patients: a read-through transcription between the neighboring BCL2L2 (BCL2-like protein 2) and PABPN1 (poliA binding protein, nuclear 1), and TBC1D3G, a GTPase activating protein for RAB5 (Figure 1). The functional annotation showed an enrichment of DE genes implicated in IL-17, TNF, NOD-like receptor, TLR receptor and NF-κβ signaling pathways (Figure 2). Regarding GO molecular functions, receptor binding, metallopeptidase activity, and calcium/zinc ion binding were the most represented terms.

### Splicing Factor Alterations in LGMDD2 Patients

Two members of the human spliceosome were differentially expressed in LGMDD2 patients. First, the calcium homeostasis endoplasmic reticulum protein (CHERP) was downregulated in LGMDD2 patients (PPDE = 0.996). On the other hand, the splicing factor 3b subunit 4 (SF3B4) was also found downregulated in LGMDD2 patients (PPDE = 0.939). The splicing targets of CHERP and SF3B4 were included in the Supplementary Table S2.

### Increased Expression of Metallopeptidases

**FIGURE 3:**
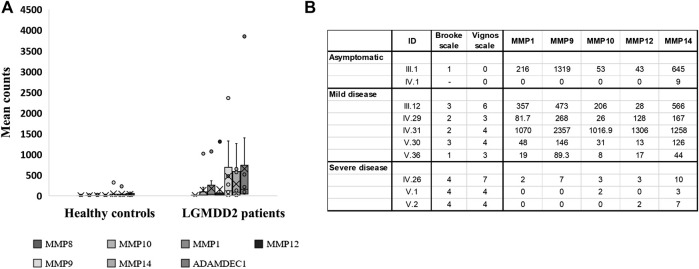
Transcripts per million of reads obtained in the RNA-Seq experiment for differentially expressed genes encoding metallopeptidases. Dashes inside boxes indicated the median value and crosses indicated the arithmetic mean. Box limits indicate the interquartile range (IQR). Whiskers are adjusted to maximal and minimal values if lower than 1.5 times the IQR. Further outliers are indicated as circles **(A)**. RNA-Seq results expressed in transcripts per million reads obtained for LGMDD2 patient according to their severity **(B)**.

Several metallopeptidases were found overexpressed in LGMDD2 patients in comparison with healthy controls, including MMP1, MMP8, MMP9, MMP10, MMP12, and MMP14 (Figure 3A). Other proteins implicated in the metallopeptidase activity and in the regulation of endopeptidase activity were also found upregulated in LGMDD2 patients, comprising ADAMTS9, ADAMTS20, ADAMDEC1, SLPI, PI3, SERPINB2, S100A8, and EPHA7.

The expression of MMP9 was clearly overexpressed in LGMDD2 patients with a mean count of 527.8 (median of transcripts per million (TPM) = 118.4; interquartile range [IQR]= 4.0–422.6) compared with the mean count of 32.5 observed in healthy controls (median TPMs = 3.0, IQR = 2.3–28.5). Within LGMDD2 group, higher levels of MMP9 (median = 268.0; IQR = 146.0–473.0) was observed in patients with mild-symptoms (understanding mild-symptoms as grades equal or lower than 3 in Brooke´s scale). On his part, low levels of MMP9 (median = 0; IQR = 0–3.5) was observed in LGMDD2 patients with severe-symptoms (associating severe symptoms with grades greater than 3 in both Brooke’s and Vignos’ scales). Statistically significant differences were found comparing the expression of MMP9 (*p* < 0.05), clearly separating mild- and severe-LGMDD2 patients according to the expression of this metallopeptidase. The same differences were observed for MMP1, MMP10, MMP12, and MMP14 (*p* < 0.05) (Figure 3B) and subsequently confirmed by qPCR (Supplementary Figure S1B).

### Enhanced Inflammatory Response in LGMDD2 Patients

**FIGURE 4:**
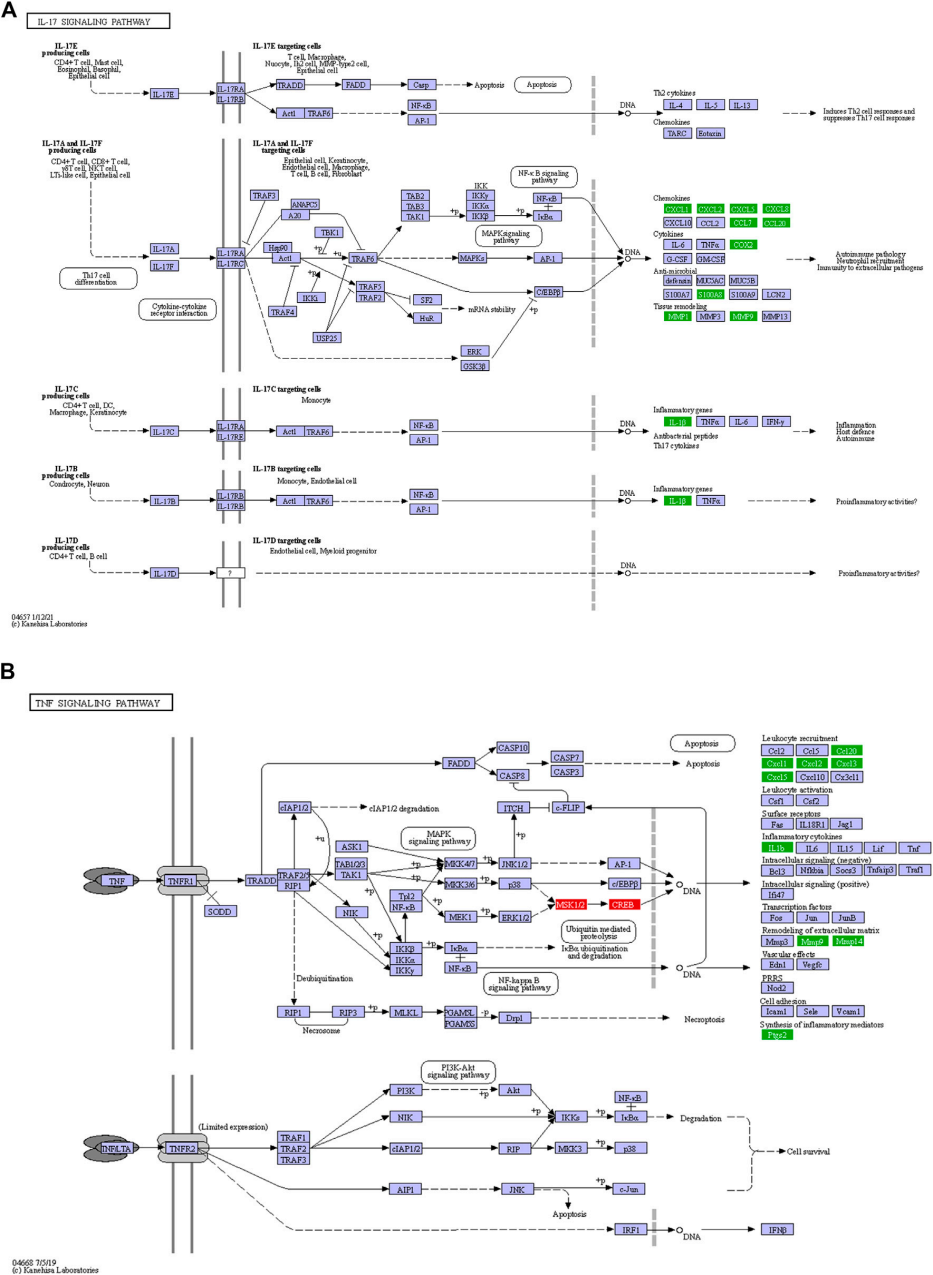
Inflammatory response in LGMDD2 patients evidenced by altered IL-17 **(A)** and TNF **(B)** signaling pathways. Green coloured genes represent overexpressed genes in LGMDD2 patients whereas red coloured genes represent genes downregulated in these individuals.

At least 12 genes related with IL-17 signaling pathway were deregulated in LGMDD2 patients. Among them, different cytokines were found overexpressed in LGMDD2 patients, including CXCL2, CXCL8, CCL20, CCL7, CXCL3, CXCL5 and CXCL1. The metallopeptidases MMP1 and MMP9 were also overexpressed in LGMDD2 patients and associated with this molecular pathway, as well as the calcium- and zinc-binding protein S100A8, the prostaglandin-endoperoxide synthase 2 (PTGS2) and the interleukin 1 beta (IL1B) (Figure 4A).

Tumor necrosis factor-α encoded by *TNF* gene is a pro-inflammatory cytokine mainly secreted by macrophages. An augmented inflammatory response in LGMDD2 patients was evidenced by a higher expression of the cytokines CXCL1, CXCL2, CXCL5 and CCL20, as well as the metallopeptidases MMP9, MMP14 and PTGS2. But also by the negative regulation of ATF6B, a transcription factor in the unfolded protein response (UPR) pathway during endoplasmic reticulum stress and RPS6KA5, a serine/threonine-protein kinase implicated in the phosphorylation of ATF subfamily members (Figure 4B).

### Exacerbated Innate Immune Response in LGMDD2 Patients

**FIGURE 5:**
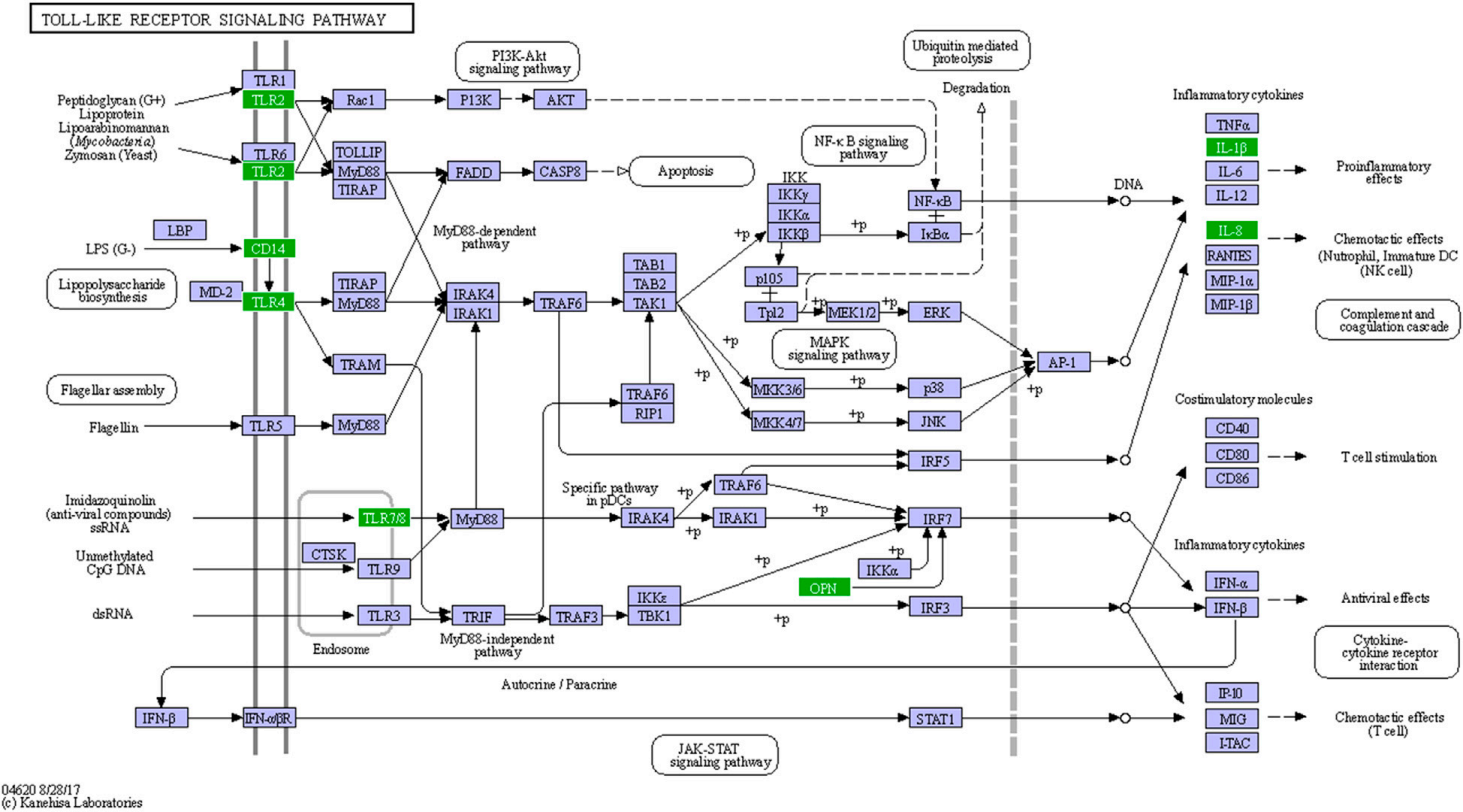
Sensing of PAMPs through TLRs in LGMDD2 patients. Green coloured genes represent overexpressed genes in LGMDD2 patients.

An enhanced innate immune response was expected in LGMDD2 patients because of the dysregulation of at least 34 genes related to this response, in comparison with healthy controls (Supplementary Table S3). Several genes included in KEGG as members of the Toll-like receptors (TLR) and NF-κB signaling pathways were also differentially expressed between LGMDD2 patients and healthy controls. Thus, TLR2, 4 and 8 were overexpressed in LGMDD2 patients along with IL1B, CD14, CXCL8 and the cytokine SPP1/Osteopontin (Figure 5), that upregulates the expression of IFNγ and IL-12, both involved in Th1 differentiation, and reduces the production of the Th2 cytokine IL-10, which leads to enhanced Th1 response.

**FIGURE 6:**
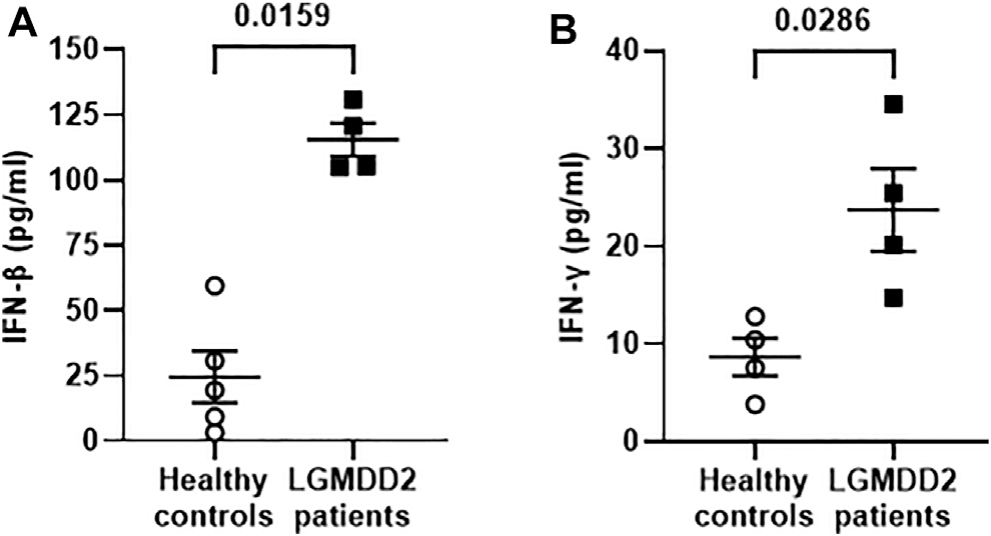
Antiviral cytokines profile in plasma samples of healthy controls and LGMDD2 patients. Levels (pg/ml) of antiviral cytokines IFNβ **(A)** and IFNγ **(B)** were quantified in plasma of healthy controls and LGMDD2 patients showing a significant increase in both IFN (p-values = 0.0159 and 0.0286, respectively).

Due to the importance of the innate immune responses in the reaction against viral infections, the expression levels of IFN-β and IFN-γ was measured in the plasma of a subgroup of seven LGMDD2 patients and seven healthy controls by Luminex assay. We observed a significant increase in the levels of IFN-β (*p* = 0.0159) (Figure 6A) and IFN-γ (*p* = 0.0286) (Figure 6B) in LGMDD2 patients (4.7- and 2.7-fold, respectively).

### Increased Expression of the Interferon-Stimulated *MxA* Gene Upon HIV-1 Infection in PBMCs From LGMDD2 Patients

**FIGURE 7:**
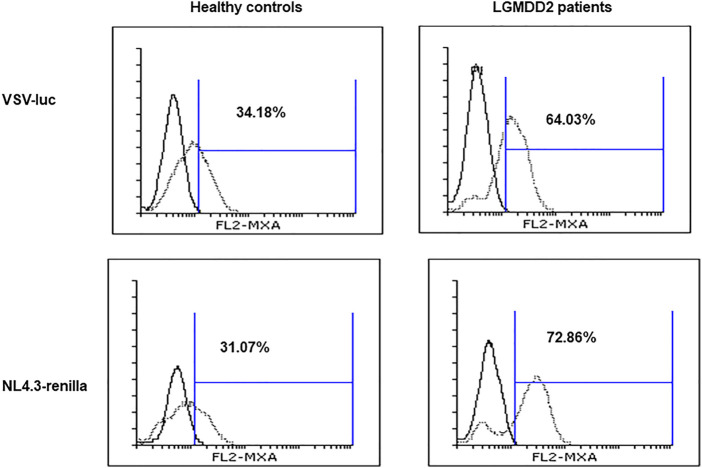
Type I interferon production. PBMCs isolated from healthy controls and LGMDD2 patients were infected by spinoculation with two different HIV-1 strains: VSV-luc and NL4-3_renilla. The production of type I IFN was measured 48 h post infection by quantifying MxA expression by flow cytometry.

Due to the resistance to HIV-1 infection described for PMBCs from LGMDD2 patients (Rodríguez-Mora et al., 2019) and the observed increase in TLR expression, we analyzed the induction of IFN-mediated responses in LGMDD2 patients as a potential antiviral mechanism. To this aim, activated PBMCs were infected *ex vivo* by spinoculation with NL4.3-Renilla or an Env-deficient HIV (NL4.3Δenv-Renilla) pseudotyped with the envelope of Stomatitis Vesicular Virus (VSV) and the intracellular expression of MxA, an IFN-stimulated gene, was measured 5 days post-infection. Average percentage of MxA expression was 1.9 and 2.3-fold higher in PMBCs from LGMDD2 patients infected with pseudotyped-VSV- and NL4.3-Renilla, respectively, in comparison with healthy controls (Figure 7). These results suggested an increase in IFN-dependent responses in PBMCs from LGMDD2 patients after HIV-1 infection, as a potential mechanism contributing to the resistance to HIV-1 infection observed in these cells.

## Discussion

As a member of the karyopherin β superfamily of proteins (Christie et al., 2016), TNPO3 imports mostly serine/arginine (SR)-rich proteins, which are essential pre-mRNA splicing factors, as SF2, SC35 and CPSF6 (Maertens et al., 2014). As the capacity to bind SR-rich domain splicing factors is located near the C-terminus of TNPO3, the additional 15 amino acids of TNPO3 observed in LGMDD2 patients could alter this cargo-binding capacity and ultimately modify the alternative splicing of mRNAs processed by factors such as CPSF6 (Jang et al., 2019). Actually, we have described (Rodríguez-Mora et al., 2019) that CPSF6 is located in both the nucleus and the cytosol of PBMCs from LGMDD2 patients, whereas it is almost exclusively nuclear in PBMCs from healthy controls. Moreover, recent crystal structures of TNPO3 showed a homodimeric form of this protein, suggesting a plausible loss of the cargo function of dimeric structures composed of the wild type and the mutated TNPO3 proteins in LGMDD2 patients (Jang et al., 2019). It is important to note that both isoforms are expressed in LGMDD2 patients (Rodríguez-Mora et al., 2019) and in this dimeric model, the mutated form of TNPO3 would act as a transdominant negative protein. To assess the potential impact of the mutation of TNPO3 in the global gene expression, we have performed a full transcriptome analysis by next-generation sequencing of PBMCs from LGMDD2 patients in comparison with healthy controls.

In LGMDD2 patients, two protein members of the human spliceosome, CHERP and SF3B4, were down-regulated. These proteins are associated with the human U2 small nuclear ribonucleoprotein (snRNA), which plays an essential role in branch point selection and catalysis during pre-mRNA splicing (Zhang et al., 2020). CHERB contains a carboxy-terminal serine/arginine-rich domain such as other splicing factors transported into the nucleus by TNPO3 (Laplante et al., 2000). Its downregulation could be caused by a defective transport of SF2 by TNPO3 because this protein is implicated in the processing of CHERP mRNA (Hyung et al., 2018). CHERP knockdown results in the inhibition of the intracellular mobilization of Ca^2+^. Regarding SF3B4, this gene encodes one of the four subunits of the splicing factor 3B and mutations in this gene have been found in families affected by Nager syndrome (Bernier et al., 2012; Czeschik et al., 2013). Strictly, SF3B4 did not reach the threshold to be considered DE but the important role of this factor in alternative splicing mechanisms deserves its discussion. Moreover, this splicing factor seems to regulate CPSF6 expression by binding to the exon 6 of CPSF6 pre-miRNA (Hyung et al., 2018).

Among the main differences observed in LGMDD2 patients, a set of genes implicated in the innate immune response and inflammation as chemokines and cytokines were upregulated, suggesting that these patients presented a basal immune activation. This increased activity could be due to a secondary inflammatory response to muscular damage. Actually, autophagy and macrophage infiltration has been found in muscle biopsies from LGMDD2 patients (Cenacchi et al., 2013; Peterle et al., 2013). However, we cannot exclude that this increased inflammatory activity could be directly related to a reduced nuclear import of splicing factors as CPSF6 due to TNPO3 mutation. Modifications in alternative polyadenylation is an important mechanism of gene expression regulation through generation of RNA isoforms with distinct 3′ termini (Martin et al., 2012; Tan et al., 2021).

The inflammatory response is not only characteristic of LGMDD2 but rather has been described in different muscular dystrophies and subtypes of LGMD (Georganopoulou et al., 2021; González-Mera et al., 2021). So much so that some non-disease-specific treatments with a therapeutic effect that reduces inflammation are being tested in patients with different muscular dystrophies, including LGMD. This is the case of a phase Ib/II study that showed that the administration of Resolaris (a histidyl tRNA synthetase stimulator) twice every week, demonstrated an improved muscle function after 12 weeks of treatment in 7 out of the 9 LGMDR2 patients included in the study (Vissing et al., 2017).

Functional analysis revealed enhanced inflammatory response in LGMDD2 patients through three interconnected mechanisms: first, an altered regulation of the IL-17 signaling pathway in the center of the pro-inflammatory response, which in turn triggers the second and third mechanisms, the TNF signaling pathway and the metallopeptidase activity. The IL-17 family of cytokines consists of 6 proteins (IL-17A to IL-17F) and 5 receptors (IL-17RA to IL-17RE) which are produced by different types of immune cells and are structurally unrelated to any other known cytokine receptor (Gaffen, 2011). Among the most important genes positively regulated by IL-17 are inflammatory cytokines, chemokines and matrix metallopeptidases (Veldhoen, 2017) that were detected in the present study. In particular, the upregulation of the chemokines CXCL1, CXCL2, CXCL5, CXCL8, CCL7, CCL20, the cytokine PTGS2 and the anti-microbial molecules S100A8 and S100A9, evidenced the activation of IL-17A/IL-17F-signaling pathways. More specifically, the induction of IL-1β observed in RNA-Seq data and IFN-γ measured by Luminex in the plasma of these patients revealed the activation of IL-17C signaling pathway, which is specific of CD4^+^ T cells, dendritic cells, macrophages and epithelial cells (Makinde et al., 2021). Interestingly, increased IFN-γ may impinge on disease atrophy through impaired myogenesis and pro-cachectic gene expression (Londhe and Davie, 2011; Ma et al., 2017).

The activation of the metallopeptidase activity through IL-17 has been demonstrated in a human pulmonary tuberculosis model, suggesting that MAPK and PI3K signaling pathways are also involved in the process (Singh et al., 2018). In transcriptome from LGMDD2 patients, IL-17 expression was correlated with an increased expression of at least 10 metallopeptidases, including MMP1, MMP8, MMP9, MMP10, MMP12, MMP14, ADAMDEC1, ADAMTS9, ADAMTS20, and CPA5. Such increase could enhance MMP-mediated processing of chemokines and contribute to the basal inflammation observed in LGMDD2 patients as these molecules are key in leukocyte trafficking and recruitment. Specifically, MMP9, MMP8, and MMP14-proteolysis of IL-8, which is also upregulated in LGMDD2 patients, may lead to an increase in its chemotactic potency by guiding the neutrophils to the site of injury or infection (Van Lint and Libert, 2007).

On the other hand, MMPs also affect the progression of inflammation by processing a variety of cytokines such as TNF, IL-1β or TGF-β (Cui et al., 2017). Actually, TNF signaling pathway and *IL1B* mRNA expression were altered in LGMDD2 patients. TNF is an important cytokine implicated in cellular processes such as apoptosis, cell survival, inflammation and regulation of the immune response. The active homotrimer of TNF binds to its main receptor TNFR1, which is expressed in nearly all cell types. TNFR1 signaling induces the activation of genes through two distinct pathways, NF-κB pathway and MAPK cascade (Daub et al., 2020). LGMDD2 patients showed an upregulation of several genes involved in this signaling pathway, including different chemokines (CXCL1, CXCL2, CXCL5 and CXCL20), inflammatory cytokines such as IL-1β, the metallopeptidases MMP9 and MMP14 implicated in remodeling the extracellular matrix and the synthesis of inflammatory mediators such as PTGS2 (Wang and Khalil, 2018). In addition, high levels of MMP9 (also in MMP1, MMP10, MMP12, and MMP14) were observed in LGMDD2 patients with mild-symptoms and compared with those with severe symptoms (which in turn, showed levels similar to those seen in controls). In this regard, it would be interesting to further investigate MMP9 and other metallopeptidases as biomarkers of disease progression, similar to what occurs in Duchenne muscular dystrophy (Nadarajah et al., 2011). Therapeutic strategies based on these proteins should also be explored for the early stages of LGMDD2, which is when the expression of these markers is characteristically high.

Finally, another indication of the potential role of basal immune activation in LGMDD2 pathology is the activation of the TLR signaling pathway. This family of receptors of 10 members (TLR1-TLR10) acts recognizing pathogen-associated molecular patterns (PAMPs) in the cell surface or in intracellular compartments. Upon recognition of PAMPs, TLRs initiate signal transduction pathways that culminate in the activation of NF-κB, IFN regulatory factors (IRFs) or MAP kinases to regulate the expression of cytokines, chemokines and type I IFNs, ultimately implicated in host protection from microbial infections (Kawai and Akira, 2010; Kawasaki and Kawai, 2014). In the present study, TLR2, TLR4 and TLR8 were found overexpressed in LGMDD2 patients along with several inflammatory cytokines such as IL-1β, IL-8, and type I IFN. These results suggest the simultaneous activation of the two branches of the TLR signaling pathway, including the MyD88-dependent pathway that leads to the production of pro-inflammatory cytokines with the activation of NFκB and MAPK, and the MyD88-independent pathway associated with the induction of IFN-inducible genes (Peterson et al., 2018; De Paepe, 2020). Enhanced IFN responses were functional in LGMDD2 as demonstrated by the overexpression of the MxA protein upon *in vitro* infection of PBMCs with HIV-1 in comparison with healthy controls.

In conclusion, although these results are mainly confirmatory about what was observed in other muscular dystrophies, the present study gives a new perspective of LGMDD2 pathology from a holistic point of view, providing the factors involved in this type of muscular dystrophy, such as IL-17, metallopeptidases, cytokines such as TNF and IL-1β or interferons, which together suggest a basal immune activation and an inflammatory state in LGMDD2 patients. Moreover, the implications of other components of the human spliceosome like CHERP and SF3B4 need to be further investigated due to their potential consequences in the general alternative splicing mechanism. These findings support our previous study demonstrating that cells from these patients are strongly resistant to HIV-1 infection and provide new clues about the antiviral underlying mechanisms. Finally, the investigation of the main pathways found altered and associated with LGMDD2 pathology has obvious implications in the design of new therapeutic strategies, not only for this type of muscular dystrophy but also for HIV-1 infection.

## Data Availability

The original contributions presented in the study are publicly available. This data can be found here: https://www.ncbi.nlm.nih.gov/geo/query/acc.cgi?acc=GSE193662.
