# Supplementary material for: Transcriptomic Evidence of the Immune Response Activation in Individuals With Limb Girdle Muscular Dystrophy Dominant 2 (LGMDD2) Contributes to Resistance to HIV-1 Infection
Source: Front Cell Dev Biol. 2022 May 13;10:839813. doi: 10.3389/fcell.2022.839813 (PMC9136291; doi:10.3389/fcell.2022.839813)
Supplement: Supplementary file 1 [file DataSheet2.PDF]

Supplementary table 2.

Interacting partner 1	Interacting partner 2	Interaction type	Score	PubMed ID
CHERP	SNRNP70	Y2H: Bidirectional	0.81	22365833
CHERP	SF3B4	Y2H: Prey/Bait	1	22365833
CHERP	U2AF1	Y2H: Bidirectional	1	22365833
CHERP	U2AF2	Pull-down	—	22365833
CHERP	U2AF2	Y2H: Prey/Bait	1	22365833
CHERP	PRPF40A	Y2H: Bidirectional	0.88	22365833
CHERP	SART1	Y2H: Bait/Prey	0.94	22365833
CHERP	PRPF38A	Y2H: Bidirectional	1	22365833
CHERP	DHX8	Y2H: Bait/Prey	1	22365833
CHERP	DHX8	Pull-down	—	22365833
CHERP	SNIP1	Y2H: Bait/Prey	1	22365833
CHERP	RNPS1	Y2H: Prey/Bait	0.75	22365833
CHERP	CCAR1	Y2H: Bidirectional	0.88	22365833
CHERP	GNB2L1	Y2H: Prey/Bait	1	22365833
CHERP	RBM7	Y2H: Prey/Bait	0.5	22365833
CHERP	RBM39	Y2H: Bait/Prey	0.5	22365833
CHERP	ARGLU1	Y2H: Bidirectional	0.7	22365833
CHERP	HNRNPH3	Y2H: Prey/Bait	1	22365833
CHERP	SNRNP27	Y2H: Prey/Bait	1	22365833
CHERP	SNRNP27	Pull-down	—	22365833
CHERP	LUC7L	Y2H: Prey/Bait	1	22365833
CHERP	LUC7L	Pull-down	—	22365833
CHERP	TTC14	Y2H: Prey/Bait	0.5	22365833
CHERP	FRA10AC1	Pull-down	—	22365833
CHERP	FRA10AC1	Y2H: Bidirectional	0.81	22365833
CHERP	AGGF1	Y2H: Bidirectional	0.81	22365833
CHERP	PPIL4	Y2H: Prey/Bait	0.5	22365833
SF3B4	SF3B2	Pull-down	—	22365833
SF3B4	SF3B2	Dervied from Literature	—	7958871, 10490618, 16189514
SF3B4	SF3B2	Y2H: Bidirectional	1	22365833
SF3B4	DDX42	Y2H: Bait/Prey	0.75	22365833
SF3B4	CHERP	Y2H: Bait/Prey	1	22365833
SF3B4	SF1	Y2H: Bait/Prey	1	22365833
SF3B4	PRPF8	Y2H: Bait/Prey	0.42	22365833
SF3B4	PRPF8	Pull-down	—	22365833
SF3B4	EFTUD2	Y2H: Bait/Prey	0.81	22365833
SF3B4	EFTUD2	Pull-down	—	22365833
SF3B4	BCAS2	Y2H: Bait/Prey	1	22365833
SF3B4	BCAS2	Pull-down	—	22365833
SF3B4	WDR83	Y2H: Bait/Prey	1	22365833
SF3B4	WDR83	Pull-down	—	22365833
SF3B4	SRSF6	Y2H: Bait/Prey	0.75	22365833
SF3B4	HNRNPUL1	Y2H: Bait/Prey	0.75	22365833
SF3B4	RBM10	Y2H: Bait/Prey	1	22365833
SF3B4	RBM10	Pull-down	—	22365833

SF3B4	DDX17	Y2H: Bait/Prey	0.75	22365833
SF3B4	ILF3	Y2H: Bait/Prey	1	22365833
SF3B4	HNRNPD	Y2H: Bait/Prey	1	22365833
SF3B4	HNRNPF	Y2H: Bait/Prey	1	22365833
SF3B4	HNRNPH1	Y2H: Bait/Prey	0.75	22365833
SF3B4	RBM4	Y2H: Bait/Prey	1	22365833
