# Supplementary figures and images for: Transcriptomic Evidence of the Immune Response Activation in Individuals With Limb Girdle Muscular Dystrophy Dominant 2 (LGMDD2) Contributes to Resistance to HIV-1 Infection
Source: Front Cell Dev Biol. 2022 May 13;10:839813. doi: 10.3389/fcell.2022.839813 (PMC9136291; doi:10.3389/fcell.2022.839813)

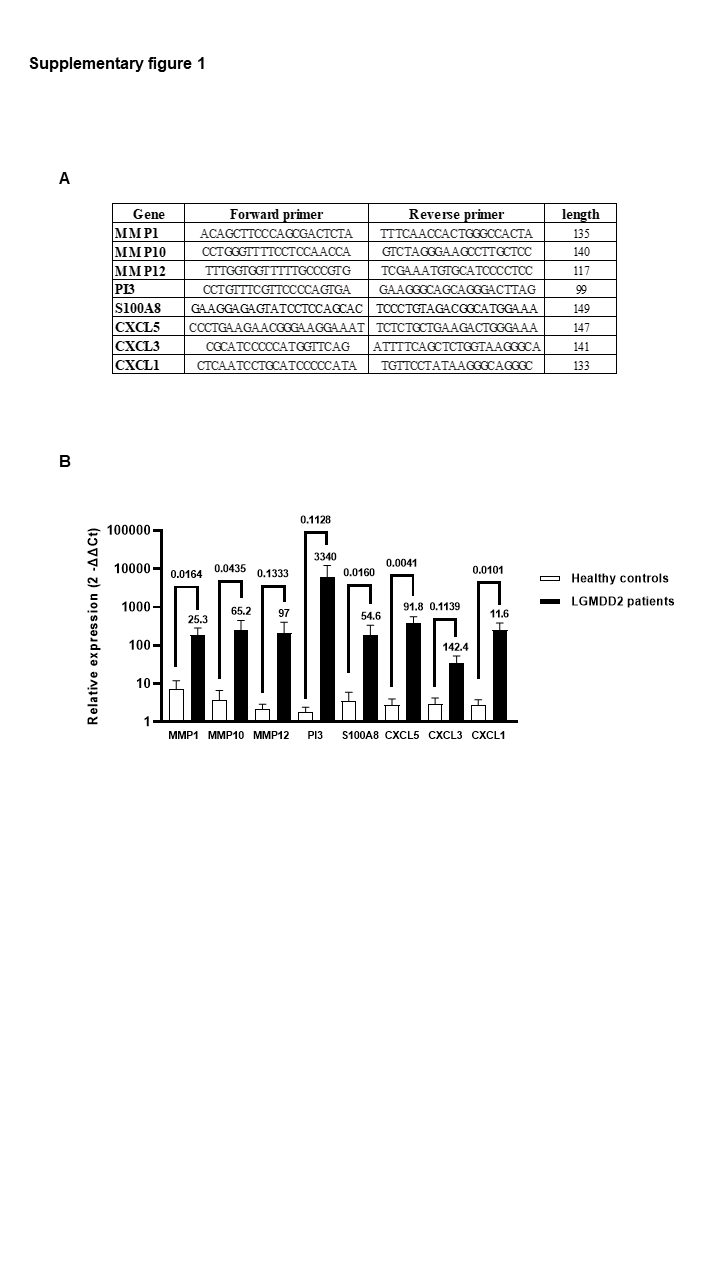

Supplement: Supplementary file 2 [file Image1.TIF]
