# Supplementary material for: Transcriptomic Evidence of the Immune Response Activation in Individuals With Limb Girdle Muscular Dystrophy Dominant 2 (LGMDD2) Contributes to Resistance to HIV-1 Infection
Source: Front Cell Dev Biol. 2022 May 13;10:839813. doi: 10.3389/fcell.2022.839813 (PMC9136291; doi:10.3389/fcell.2022.839813)
Supplement: Supplementary file 3 [file DataSheet3.PDF]

<u>Supplementary table 3.</u>

ID	PPDE	FC	Healthy controls mean	LGMDD2 patients mean
**CLEC5A**	0.998	0.046	22.427	482.862
**TLR4**	0.983	0.113	6.990	61.915
**CLEC6A**	0.994	0.061	6.752	111.451
**TLR2**	0.995	0.105	40.261	383.507
**S100A8**	1.000	0.009	1.332	148.033
**HLA-DQA2**	0.953	3.782	75.794	20.032
**PUM2**	0.958	1.410	3420.849	2425.903
**TGFB1**	0.973	1.378	389.031	282.235
**TNIP3**	0.998	0.531	614.484	1158.096
**C1QB**	0.967	0.090	1.645	18.387
**C1QC**	0.992	0.059	0.525	9.072
**CD300E**	1.000	0.043	12.205	287.141
**NLRP3**	0.989	0.126	15.580	124.184
**CTSL**	1.000	0.069	92.599	1348.780
**MARCO**	1.000	0.011	0.060	6.172
**VNN1**	0.990	0.123	1.034	8.462
**MT2A**	0.989	0.531	2265.609	4269.876
**CCL8**	1.000	0.036	4.075	113.989
**LEP**	0.975	0.121	2.906	24.056
**CD14**	0.999	0.068	5.421	79.748
**CCL20**	1.000	0.150	24.443	163.083
**RAB27A**	1.000	1.697	1483.336	874.230
**WDFY1**	0.975	0.601	1169.825	1945.019
**HLA-G**	0.978	2.204	139.862	63.459
**FCGR1A**	0.998	0.066	9.899	149.048
**CCL7**	1.000	0.001	0.000	9.367
**TREM1**	1.000	0.037	6.418	173.368
**TMEM189-UBE2V1**	0.992	7.260	8.560	1.171
**RPS6KA5**	0.993	1.589	1631.753	1027.161
**S100A9**	1.000	0.048	9.092	190.917
**S100A12**	1.000	0.009	0.098	12.665
**TLR8**	0.973	0.095	19.281	203.912
**CLEC4D**	0.973	0.074	3.571	48.404
**SLPI**	1.000	0.001	0.000	19.630
