# Supplementary material for: Transcriptomic Evidence of the Immune Response Activation in Individuals With Limb Girdle Muscular Dystrophy Dominant 2 (LGMDD2) Contributes to Resistance to HIV-1 Infection
Source: Front Cell Dev Biol. 2022 May 13;10:839813. doi: 10.3389/fcell.2022.839813 (PMC9136291; doi:10.3389/fcell.2022.839813)
Supplement: Supplementary file 4 [file DataSheet1.PDF]

Supplementary table 1.

ID	PPDE	RealFC	Healthy controls	LGMDD2 patients	Ensembl ID2	log2 FC
**SLPI**	1.000	0.001	0.000	19.630	ENSG00000124107	-10.940
**PI3**	1.000	0.001	0.110	231.225	ENSG00000124102	-10.911
**MMP8**	1.000	0.001	0.000	10.255	ENSG00000118113	-10.004
**CCL7**	1.000	0.001	0.000	9.367	ENSG00000108688	-9.873
**TRGV8**	1.000	0.001	0.000	6.948	ENSG00000211696	-9.443
**TMEM26**	0.998	0.002	0.000	6.362	ENSG00000196932	-9.316
**FAM138F**	1.000	0.002	0.000	5.075	ac	-8.990
**SERPINB2**	1.000	0.006	0.226	40.135	ENSG00000197632	-7.413
**SLAMF9**	1.000	0.008	0.181	23.565	ENSG00000162723	-6.947
**S100A12**	1.000	0.009	0.098	12.665	ENSG00000163221	-6.876
**S100A8**	1.000	0.009	1.332	148.033	ENSG00000143546	-6.785
**CLVS2**	1.000	0.011	0.060	6.625	ENSG00000146352	-6.559
**MARCO**	1.000	0.011	0.060	6.172	ENSG00000019169	-6.458
**MMP10**	1.000	0.012	2.030	176.798	ENSG00000166670	-6.438
**EPHA5**	0.999	0.013	0.060	5.276	ENSG00000145242	-6.232
**MMP1**	1.000	0.014	3.052	214.928	ENSG00000196611	-6.133
**PWRN2**	0.999	0.019	0.081	4.808	ENSG00000260551	-5.733
**CXCL5**	1.000	0.019	7.868	407.712	ENSG00000163735	-5.694
**MMP12**	1.000	0.020	4.276	211.965	ENSG00000262406	-5.628
**ADGRE3**	1.000	0.022	0.109	5.388	ENSG00000131355	-5.503
**EPHA7**	0.996	0.024	0.081	3.749	ENSG00000135333	-5.375
**CXCL3**	1.000	0.026	4.300	165.084	ENSG00000163734	-5.259
**CXCL1**	1.000	0.026	7.664	291.435	ENSG00000163739	-5.247
**UNC5D**	0.997	0.028	0.121	4.723	ENSG00000156687	-5.178
**ADGRL2**	1.000	0.028	0.158	5.954	ENSG00000117114	-5.148
**RP4-737E23.6**	0.998	0.030	0.118	4.324	ENSG00000279447	-5.081
**RP11-3N2.8**	0.968	0.030	0.060	2.370	ENSG00000227035	-5.080
**AC096559.1**	0.968	0.030	0.060	2.344	ENSG00000224184	-5.064
**ADAMTS20**	0.995	0.030	0.098	3.540	ENSG00000173157	-5.040
**PTPRQ**	1.000	0.031	0.157	5.381	ENSG00000139304	-5.011
**RP11-403B2.6**	1.000	0.032	0.194	6.460	ENSG00000259383	-4.985
**RP11-1008C21.1**	1.000	0.032	0.606	19.133	ENSG00000259225	-4.957
**ABLIM3**	0.997	0.033	0.098	3.294	ENSG00000173210	-4.937
**LINC01127**	0.998	0.033	0.278	8.720	ENSG00000281162	-4.923
**ADGRL3**	1.000	0.034	0.205	6.389	ENSG00000150471	-4.898
**RP11-766F14.2**	0.951	0.034	0.081	2.689	ENSG00000248713	-4.897
**VCAN**	1.000	0.034	22.685	673.083	ENSG00000038427	-4.890
**PAK7**	0.993	0.034	0.110	3.500	ENSG00000101349	-4.869
**RP11-290D2.6**	1.000	0.034	0.201	6.122	ENSG00000273149	-4.863
**PDPN**	1.000	0.034	0.793	23.319	ENSG00000162493	-4.860
**SPP1**	1.000	0.035	21.255	607.399	ENSG00000118785	-4.836
**CCL8**	1.000	0.036	4.075	113.989	ENSG00000108700	-4.803
**CA12**	1.000	0.036	10.882	303.147	ENSG00000074410	-4.799
**RNASE1**	1.000	0.036	0.540	15.092	ENSG00000129538	-4.780
**RPL23AP25**	0.997	0.036	0.111	3.300	ENSG00000233084	-4.779
**THBD**	1.000	0.037	2.991	82.130	ENSG00000178726	-4.775

**CXCL8**	1.000	0.037	480.943	13155.566	ENSG00000169429	-4.774
**POTEH-AS1**	0.996	0.037	0.181	5.179	ENSG00000236666	-4.763
**AC092684.1**	1.000	0.037	0.609	16.696	ENSG00000237844	-4.754
**TREM1**	1.000	0.037	6.418	173.368	ENSG00000124731	-4.753
**RP5-907C10.3**	0.956	0.037	0.081	2.406	ENSG00000279419	-4.737
**FAM225A**	0.998	0.038	0.606	16.213	ENSG00000231528	-4.719
**ERBB4**	0.998	0.038	0.167	4.624	ENSG00000178568	-4.709
**RGS7BP**	0.961	0.039	0.081	2.340	ENSG00000186479	-4.697
**CDH19**	0.996	0.039	0.152	4.117	ENSG00000071991	-4.671
**CTD-2545G14.7**	1.000	0.040	3.059	77.407	ENSG00000262526	-4.657
**ADAMDEC1**	1.000	0.042	32.933	778.164	ENSG00000134028	-4.562
**CD300E**	1.000	0.043	12.205	287.141	ENSG00000186407	-4.555
**ANO3**	0.978	0.043	0.121	3.014	ENSG00000134343	-4.532
**C8orf48**	0.992	0.043	0.132	3.265	ENSG00000164743	-4.532
**CD163**	1.000	0.044	4.503	103.214	ENSG00000177575	-4.515
**CFAP47**	0.995	0.044	0.132	3.219	ENSG00000165164	-4.512
**RP11-725P16.2**	0.979	0.044	0.107	2.636	ENSG00000272769	-4.502
**AC112198.1**	0.983	0.044	0.170	4.077	ENSG00000232517	-4.502
**FPR1**	1.000	0.045	1.537	34.295	ENSG00000171051	-4.471
**MUC7**	0.955	0.045	0.081	1.988	ENSG00000171195	-4.463
**EEF1DP5**	0.969	0.046	0.098	2.353	ENSG00000213130	-4.453
**LILRA1**	1.000	0.046	3.184	69.842	ENSG00000104974	-4.451
**CLEC5A**	0.998	0.046	22.427	482.862	ENSG00000258227	-4.428
**RP11-707A18.1**	0.982	0.047	0.170	3.852	ENSG00000250125	-4.420
**S100A9**	1.000	0.048	9.092	190.917	ENSG00000163220	-4.391
**IL1B**	1.000	0.048	151.236	3163.581	ENSG00000125538	-4.387
**SLC22A2**	0.999	0.048	0.206	4.504	ENSG00000112499	-4.387
**KCNJ2**	1.000	0.049	3.721	75.619	ENSG00000123700	-4.341
**HCAR3**	1.000	0.050	3.060	61.381	ENSG00000255398	-4.322
**CXCL2**	1.000	0.050	7.032	140.072	ENSG00000081041	-4.314
**HCAR2**	0.999	0.050	6.373	126.412	ENSG00000182782	-4.308
**TBX18**	0.993	0.052	0.157	3.204	ENSG00000112837	-4.265
**KRT23**	0.978	0.052	0.387	7.604	ENSG00000108244	-4.262
**UGT2B11**	0.965	0.054	0.118	2.369	ENSG00000213759	-4.216
**RP11-81A1.7**	0.950	0.055	0.351	6.621	ENSG00000279719	-4.197
**SLITRK4**	0.999	0.055	0.601	11.051	ENSG00000179542	-4.179
**FAM230B**	0.990	0.057	0.216	3.989	ENSG00000215498	-4.146
**SLC5A1**	0.960	0.057	0.132	2.495	ENSG00000100170	-4.145
**PSG9**	0.998	0.058	0.233	4.179	ENSG00000183668	-4.110
**CASP5**	1.000	0.058	1.330	22.966	ENSG00000137757	-4.099
**CTD-2006K23.1**	0.990	0.058	0.279	4.945	ENSG00000261222	-4.099
**LYZ**	0.980	0.059	479.399	8167.749	ENSG00000090382	-4.091
**C1QC**	0.992	0.059	0.525	9.072	ENSG00000159189	-4.085
**CES1**	0.995	0.060	0.514	8.676	ENSG00000198848	-4.051
**CLEC6A**	0.994	0.061	6.752	111.451	ENSG00000205846	-4.043
**KCNA6**	0.983	0.061	0.301	5.084	ENSG00000151079	-4.036
**AC009541.1**	0.975	0.061	0.152	2.647	ENSG00000226066	-4.036
**FLRT3**	0.984	0.061	0.192	3.288	ENSG00000125848	-4.030
**MMP9**	1.000	0.062	32.451	527.773	ENSG00000100985	-4.023
**MYOCD**	0.965	0.062	0.249	4.198	ENSG00000141052	-4.022

**RNF17**	1.000	0.062	4.337	70.057	ENSG00000132972	-4.011
**ADAMTS9**	0.999	0.062	0.357	5.877	ENSG00000163638	-4.005
**COL6A5**	0.997	0.062	0.267	4.432	ENSG00000172752	-4.002
**RP11-386M24.6**	0.980	0.063	0.187	3.127	ENSG00000260337	-3.991
**TNFAIP6**	1.000	0.064	8.781	137.113	ENSG00000123610	-3.963
**OR2I1P**	0.999	0.065	0.636	9.979	ENSG00000237988	-3.951
**TIFAB**	0.966	0.065	0.381	6.039	ENSG00000255833	-3.951
**PRRX1**	0.993	0.065	0.216	3.464	ENSG00000116132	-3.944
**SLAMF8**	1.000	0.065	11.100	170.099	ENSG00000158714	-3.937
**MEI4**	0.968	0.066	0.152	2.448	ENSG00000269964	-3.924
**MPDZ**	0.999	0.066	0.442	6.817	ENSG00000107186	-3.916
**FCGR1A**	0.998	0.066	9.899	149.048	ENSG00000150337	-3.911
**FCGR2A**	1.000	0.067	79.555	1196.386	ENSG00000143226	-3.910
**PNMA6A**	1.000	0.067	0.336	5.148	ENSG00000235961	-3.899
**TM4SF1**	1.000	0.067	1.275	19.075	ENSG00000169908	-3.893
**FPR3**	0.994	0.067	8.442	125.245	ENSG00000187474	-3.889
**CD14**	0.999	0.068	5.421	79.748	ENSG00000170458	-3.876
**CTSL**	1.000	0.069	92.599	1348.780	ENSG00000135047	-3.864
**SORCS1**	0.992	0.069	0.278	4.180	ENSG00000108018	-3.863
**ACSS3**	0.970	0.069	0.386	5.733	ENSG00000111058	-3.859
**GABRA4**	0.998	0.069	0.490	7.213	ENSG00000109158	-3.852
**FGF14**	0.999	0.070	0.497	7.263	ENSG00000102466	-3.842
**NRXN1**	0.997	0.070	0.644	9.299	ENSG00000179915	-3.831
**MT1H**	1.000	0.071	1.677	23.770	ENSG00000205358	-3.817
**GOLGA8EP**	0.999	0.071	0.415	5.979	ENSG00000175676	-3.816
**USP17L15**	0.999	0.071	0.355	5.126	ENSG00000223569	-3.815
**MT1M**	0.990	0.073	0.494	6.944	ENSG00000205364	-3.786
**TBC1D3**	1.000	0.074	2.875	39.067	ENSG00000274611	-3.760
**CLEC4D**	0.973	0.074	3.571	48.404	ENSG00000166527	-3.757
**GLUD1P8**	0.999	0.075	0.602	8.191	ENSG00000265630	-3.744
**HK3**	0.967	0.075	3.899	52.242	ENSG00000160883	-3.741
**ABC7-42404400C24.1**	0.998	0.077	0.381	5.092	ENSG00000277758	-3.705
**LRRIQ1**	0.996	0.077	0.509	6.754	ENSG00000133640	-3.705
**RP11-864N7.2**	1.000	0.077	7.446	96.490	ENSG00000227615	-3.694
**RP11-283G6.4**	0.956	0.078	0.517	6.736	ENSG00000256234	-3.677
**RP11-925D8.2**	0.968	0.078	0.368	4.819	ENSG00000259626	-3.677
**TFPI2**	1.000	0.078	6.072	77.627	ENSG00000105825	-3.674
**CTB-61M7.2**	0.993	0.081	0.822	10.254	ENSG00000268734	-3.625
**LINC00472**	0.953	0.081	0.217	2.786	ENSG00000233237	-3.623
**SLITRK6**	0.950	0.081	0.277	3.526	ENSG00000184564	-3.622
**RP11-15J10.8**	0.981	0.083	0.278	3.473	ENSG00000236252	-3.595
**KRT17P1**	1.000	0.084	1.759	21.172	ENSG00000131885	-3.582
**CSRNP3**	0.993	0.084	0.643	7.744	ENSG00000178662	-3.570
**RP11-476D10.1**	0.980	0.085	0.601	7.175	ENSG00000260943	-3.555
**TDRD6**	0.998	0.085	2.606	30.660	ENSG00000180113	-3.551
**TBC1D3C**	1.000	0.086	1.313	15.449	ENSG00000278299	-3.547
**SUCNR1**	0.991	0.086	1.945	22.806	ENSG00000198829	-3.545
**LHX9**	0.999	0.086	0.598	7.091	ENSG00000143355	-3.545
**RNA5SP202**	0.952	0.086	0.550	6.517	ENSG00000201185	-3.542
**WISP1**	0.972	0.087	0.641	7.503	ENSG00000104415	-3.529

**MIR1248**	0.999	0.087	7.801	89.658	ENSG00000281017	-3.521
**GALNTL6**	0.998	0.087	0.470	5.492	ENSG00000174473	-3.518
**TGFBI**	0.998	0.088	3.527	40.263	ENSG00000120708	-3.509
**PCSK5**	1.000	0.088	196.872	2240.732	ENSG00000099139	-3.509
**BMS1P7**	0.989	0.088	0.375	4.347	ENSG00000270025	-3.501
**CYP19A1**	0.992	0.089	0.487	5.567	ENSG00000137869	-3.489
**RP11-142A12.1**	0.999	0.089	33.213	372.527	ENSG00000260580	-3.487
**AC011343.1**	0.970	0.090	0.263	3.035	ENSG00000253311	-3.479
**C1QB**	0.967	0.090	1.645	18.387	ENSG00000173369	-3.475
**CLLU1OS**	1.000	0.090	2.499	27.877	ENSG00000205057	-3.474
**PTGS2**	1.000	0.092	62.293	678.347	ENSG00000073756	-3.445
**RP11-1086I4.2**	0.977	0.092	0.326	3.637	ENSG00000279674	-3.440
**HEPH**	0.981	0.092	0.383	4.251	ENSG00000089472	-3.439
**PLCXD3**	0.970	0.092	1.135	12.378	ENSG00000182836	-3.436
**CYP26A1**	0.997	0.094	0.414	4.521	ENSG00000095596	-3.417
**RGS8**	0.964	0.094	0.265	2.914	ENSG00000135824	-3.410
**TLR8**	0.973	0.095	19.281	203.912	ENSG00000101916	-3.402
**FCGR2B**	0.990	0.095	41.419	436.101	ENSG00000072694	-3.396
**TUSC7**	0.951	0.096	0.259	2.788	ENSG00000243197	-3.380
**FCGR2C**	0.983	0.098	37.516	384.574	ENSG00000244682	-3.357
**MMP14**	0.993	0.098	30.470	310.049	ENSG00000157227	-3.347
**CTNNA3**	0.998	0.099	0.739	7.542	ENSG00000183230	-3.335
**MAB21L1**	0.980	0.102	0.360	3.627	ENSG00000180660	-3.298
**CD93**	0.999	0.103	26.331	255.024	ENSG00000125810	-3.275
**NOG**	1.000	0.103	3.361	32.596	ENSG00000183691	-3.274
**TWF1P1**	0.954	0.104	1.088	10.593	ENSG00000178082	-3.272
**LILRB4**	0.972	0.104	38.395	370.712	ENSG00000186818	-3.271
**TLR2**	0.995	0.105	40.261	383.507	ENSG00000137462	-3.251
**MDGA2**	0.977	0.105	0.622	5.989	ENSG00000139915	-3.247
**FAM216B**	0.978	0.106	0.598	5.736	ENSG00000179813	-3.241
**MS4A4A**	0.995	0.107	0.712	6.764	ENSG00000110079	-3.229
**CD300LF**	0.956	0.107	2.613	24.498	ENSG00000186074	-3.224
**PCDH15**	0.971	0.108	0.590	5.517	ENSG00000150275	-3.205
**SDC2**	0.961	0.109	26.354	241.968	ENSG00000169439	-3.198
**CH17-132F21.5**	0.992	0.110	2.149	19.687	ENSG00000281904	-3.190
**KCNH7**	0.952	0.110	0.377	3.515	ENSG00000184611	-3.187
**C15orf48**	0.975	0.112	15.512	137.980	ENSG00000166920	-3.152
**TLR4**	0.983	0.113	6.990	61.915	ENSG00000136869	-3.145
**PCLO**	0.992	0.113	1.081	9.616	ENSG00000186472	-3.141
**ARHGEF10L**	0.954	0.114	3.495	30.865	ENSG00000074964	-3.139
**CH17-270A2.2**	0.998	0.116	0.901	7.873	ENSG00000280778	-3.113
**GOLGA8CP**	1.000	0.116	2.048	17.722	ENSG00000181984	-3.107
**GRIA1**	0.970	0.119	0.386	3.324	ENSG00000155511	-3.075
**GRIN2B**	0.967	0.119	1.623	13.740	ENSG00000273079	-3.074
**NAP1L4P3**	0.966	0.120	0.369	3.146	ENSG00000234145	-3.059
**LEP**	0.975	0.121	2.906	24.056	ENSG00000174697	-3.045
**PDE10A**	0.951	0.122	0.575	4.770	ENSG00000112541	-3.031
**RP11-105C19.2**	0.970	0.123	0.579	4.793	ENSG00000260973	-3.027
**TBC1D3D**	0.977	0.123	0.849	6.967	ENSG00000274419	-3.022
**VNN1**	0.990	0.123	1.034	8.462	ENSG00000112299	-3.021

**GALNT13**	0.958	0.123	0.748	6.132	ENSG00000144278	-3.019
**TACSTD2**	0.965	0.123	0.877	7.178	ENSG00000184292	-3.019
**WASH7P**	1.000	0.125	3.079	24.695	ENSG00000227232	-2.999
**NLRP3**	0.989	0.126	15.580	124.184	ENSG00000162711	-2.994
**AF015262.2**	0.991	0.127	0.872	6.960	ENSG00000234703	-2.982
**TREML4**	0.975	0.130	0.714	5.541	ENSG00000188056	-2.939
**CP**	0.973	0.132	13.487	101.929	ENSG00000047457	-2.917
**AZGP1P1**	0.979	0.133	0.570	4.359	ENSG00000214313	-2.913
**DOCK4-AS1**	0.963	0.133	2.820	21.230	ENSG00000225572	-2.908
**CHST15**	0.972	0.136	23.988	175.903	ENSG00000182022	-2.874
**F3**	0.951	0.137	15.433	113.012	ENSG00000117525	-2.872
**ADGRF2**	0.982	0.137	4.917	35.872	ENSG00000164393	-2.865
**RP11-586K12.2**	0.983	0.142	3.973	28.001	ENSG00000279780	-2.814
**ROBO2**	0.996	0.144	1.698	11.883	ENSG00000185008	-2.800
**FOXP2**	0.982	0.145	1.254	8.729	ENSG00000128573	-2.789
**RP11-977B10.2**	0.961	0.145	0.688	4.795	ENSG00000258101	-2.784
**CCL20**	1.000	0.150	24.443	163.083	ENSG00000115009	-2.738
**ANKRD1**	0.997	0.150	2.873	19.148	ENSG00000148677	-2.732
**TMSB15A**	0.955	0.151	0.612	4.123	ENSG00000158164	-2.732
**STAC**	0.964	0.151	1.025	6.852	ENSG00000144681	-2.730
**RP11-575F12.3**	0.982	0.155	0.804	5.244	ENSG00000278266	-2.690
**KCNIP4-IT1**	0.992	0.155	1.357	8.796	ENSG00000280650	-2.687
**RP3-467K16.2**	0.997	0.159	0.894	5.662	ENSG00000233485	-2.650
**ANGPTL4**	0.982	0.159	6.636	41.661	ENSG00000167772	-2.649
**RP4-583P15.14**	0.989	0.161	1.093	6.859	ENSG00000273047	-2.639
**CTD-2144E22.9**	0.981	0.162	10.488	64.723	ENSG00000279165	-2.624
**MET**	0.974	0.163	23.548	144.817	ENSG00000105976	-2.620
**SNORD116-27**	0.960	0.164	0.545	3.379	ENSG00000251896	-2.610
**CLLU1**	0.996	0.165	6.313	38.219	ENSG00000257127	-2.596
**SLC26A9**	0.959	0.169	0.737	4.420	ENSG00000174502	-2.569
**ZNF705A**	0.952	0.171	0.767	4.543	ENSG00000196946	-2.551
**C2orf72**	0.999	0.171	6.184	36.256	ENSG00000204128	-2.550
**ADM**	0.988	0.171	34.282	200.198	ENSG00000148926	-2.546
**MARK1**	0.959	0.173	1.151	6.701	ENSG00000116141	-2.531
**RP11-406A9.2**	0.956	0.177	0.652	3.735	ENSG00000258743	-2.501
**CYS1**	0.995	0.184	1.438	7.841	ENSG00000205795	-2.438
**CA3**	0.961	0.185	0.865	4.718	ENSG00000164879	-2.434
**IGKV1D-13**	0.970	0.185	1.635	8.858	ENSG00000276566	-2.431
**LDHC**	0.985	0.187	3.774	20.176	ENSG00000166796	-2.415
**BARX1**	0.993	0.192	4.780	24.904	ENSG00000131668	-2.379
**PRR13P5**	0.979	0.199	1.247	6.321	ENSG00000187534	-2.333
**CYP4F29P**	0.976	0.219	2.281	10.429	ENSG00000228314	-2.188
**DHDH**	0.971	0.222	0.892	4.047	ENSG00000104808	-2.170
**NRN1**	0.991	0.226	29.137	129.103	ENSG00000124785	-2.147
**CLDN1**	0.979	0.228	6.417	28.136	ENSG00000163347	-2.131
**RP11-303E16.7**	0.953	0.230	1.161	5.072	ENSG00000245059	-2.117
**MT1JP**	0.960	0.236	0.812	3.477	ENSG00000255986	-2.085
**FMN1**	0.984	0.238	62.757	264.269	ENSG00000248905	-2.074
**IGFBP2**	0.974	0.246	42.348	172.128	ENSG00000115457	-2.023
**LINC00282**	1.000	0.252	2.708	10.775	ENSG00000281106	-1.989

**EPCAM**	0.999	0.281	2.146	7.676	ENSG00000119888	-1.834
**NFIX**	0.994	0.298	9.699	32.532	ENSG00000008441	-1.745
**NUDT12**	1.000	0.306	26.155	85.425	ENSG00000112874	-1.707
**CTD-2001C12.1**	0.997	0.322	2.521	7.855	ENSG00000247877	-1.636
**OCIAD1-AS1**	0.980	0.327	3.775	11.571	ENSG00000248256	-1.614
**RP5-1148A21.3**	0.999	0.329	3.206	9.755	ENSG00000266680	-1.602
**RP11-95O2.1**	0.967	0.363	3.053	8.420	ENSG00000267651	-1.460
**RCN1P2**	0.967	0.380	13.976	36.768	ENSG00000214455	-1.395
**AC017104.6**	0.994	0.385	4.890	12.708	ENSG00000224376	-1.376
**FXYD2**	1.000	0.403	7.571	18.814	ENSG00000137731	-1.312
**TCEA3**	0.999	0.424	155.439	367.040	ENSG00000204219	-1.240
**TRBV6-6**	0.996	0.458	29.760	64.991	ENSG00000211724	-1.127
**MYO5C**	0.987	0.459	83.914	182.976	ENSG00000128833	-1.125
**AC083843.1**	0.991	0.465	108.287	232.730	ENSG00000259820	-1.104
**C1orf228**	0.999	0.508	105.305	207.365	ENSG00000198520	-0.978
**RP11-98J23.2**	0.960	0.513	42.940	83.703	ENSG00000198237	-0.963
**CTD-2302E22.4**	0.975	0.514	7.505	14.615	ENSG00000261242	-0.961
**TNIP3**	0.998	0.531	614.484	1158.096	ENSG00000050730	-0.914
**MT2A**	0.989	0.531	2265.609	4269.876	ENSG00000125148	-0.914
**PKIA**	0.960	0.535	240.029	448.742	ENSG00000171033	-0.903
**TRBV20-1**	0.998	0.570	100.770	176.691	ENSG00000211747	-0.810
**SARDH**	0.982	0.573	13.892	24.253	ENSG00000123453	-0.804
**ZNF429**	1.000	0.574	235.887	411.214	ENSG00000197013	-0.802
**CLN5**	0.990	0.577	69.449	120.323	ENSG00000102805	-0.793
**EPHA1**	0.975	0.583	45.131	77.418	ENSG00000146904	-0.778
**LIPT2**	0.983	0.601	24.195	40.276	ENSG00000175536	-0.735
**WDFY1**	0.975	0.601	1169.825	1945.019	ENSG00000085449	-0.733
**NAB2**	0.961	0.606	245.388	405.124	ENSG00000166886	-0.723
**ZDBF2**	0.996	0.606	613.900	1012.281	ENSG00000204186	-0.722
**CD69**	0.968	0.610	1671.854	2741.253	ENSG00000110848	-0.713
**STAG3L4**	0.998	0.637	138.141	216.949	ENSG00000106610	-0.651
**TAF9B**	0.995	0.640	606.502	948.333	ENSG00000187325	-0.645
**LDLRAP1**	0.990	0.642	125.056	194.679	ENSG00000157978	-0.638
**CERS6-AS1**	0.988	0.646	115.707	179.228	ENSG00000227617	-0.631
**PMFBP1**	0.951	0.648	34.645	53.449	ENSG00000118557	-0.625
**IL10RB**	1.000	0.650	367.847	565.874	ENSG00000243646	-0.621
**RAB8B**	1.000	0.661	2862.583	4331.032	ENSG00000166128	-0.597
**NRIP1**	0.992	0.668	924.054	1383.521	ENSG00000180530	-0.582
**OXNAD1**	0.978	0.676	1845.590	2731.526	ENSG00000154814	-0.566
**LMBRD1**	1.000	0.685	477.229	697.141	ENSG00000168216	-0.547
**SAYSD1**	1.000	0.708	292.726	413.680	ENSG00000112167	-0.499
**PLEKHF2**	0.998	0.716	368.913	514.964	ENSG00000175895	-0.481
**STK17B**	0.992	0.720	16049.599	22293.984	ENSG00000081320	-0.474
**UGCG**	0.998	0.724	607.308	838.832	ENSG00000148154	-0.466
**CHMP7**	0.997	0.729	655.036	899.040	ENSG00000147457	-0.457
**ADAT2**	1.000	0.732	484.653	661.942	ENSG00000189007	-0.450
**CD247**	0.995	0.739	2058.867	2787.458	ENSG00000198821	-0.437
**PM20D2**	1.000	0.748	559.999	748.513	ENSG00000146281	-0.419
**HIBADH**	0.979	0.752	229.693	305.573	ENSG00000106049	-0.412
**PTEN**	0.999	0.752	2608.557	3469.210	ENSG00000171862	-0.411

**STK38L**	0.993	0.761	859.483	1129.561	ENSG00000211455	-0.394
**UMAD1**	0.951	0.780	274.881	352.496	ENSG00000219545	-0.359
**SMIM10L1**	1.000	0.792	364.472	460.315	ENSG00000256537	-0.337
**SIRT5**	0.986	0.802	245.414	305.999	ENSG00000124523	-0.318
**ARL14EP**	0.995	0.810	687.756	849.326	ENSG00000152219	-0.304
**CHD6**	1.000	0.811	1959.748	2416.548	ENSG00000124177	-0.302
**STX6**	0.995	0.819	914.498	1117.236	ENSG00000135823	-0.289
**BBS4**	0.979	0.843	215.974	256.052	ENSG00000140463	-0.246
**SNX1**	0.989	1.182	900.199	761.513	ENSG00000028528	0.241
**NBEAL2**	0.965	1.188	1095.799	922.557	ENSG00000160796	0.248
**CERS5**	1.000	1.223	421.248	344.368	ENSG00000139624	0.291
**C10orf76**	0.983	1.228	282.717	230.213	ENSG00000120029	0.296
**DNM2**	0.966	1.237	2114.137	1709.093	ENSG00000079805	0.307
**FLYWCH1**	0.982	1.248	405.553	325.058	ENSG00000059122	0.319
**PDP2**	0.996	1.259	696.975	553.708	ENSG00000172840	0.332
**CYTH2**	0.999	1.281	498.119	388.926	ENSG00000105443	0.357
**ARHGAP35**	1.000	1.296	1434.179	1107.033	ENSG00000160007	0.374
**SLC27A5**	0.991	1.303	278.407	213.658	ENSG00000083807	0.382
**TP53**	0.986	1.308	1005.705	768.819	ENSG00000141510	0.387
**PPP3R1**	0.997	1.314	1088.436	828.158	ENSG00000221823	0.394
**EMC3-AS1**	0.961	1.335	178.734	133.833	ENSG00000180385	0.417
**SLC25A30**	1.000	1.338	563.647	421.193	ENSG00000174032	0.420
**ARID3B**	0.989	1.356	1177.567	868.602	ENSG00000179361	0.439
**GUSB**	0.998	1.359	414.306	304.804	ENSG00000169919	0.443
**TGFB1**	0.973	1.378	389.031	282.235	ENSG00000105329	0.463
**ATF6B**	0.977	1.383	737.534	533.114	ENSG00000213676	0.468
**CD81**	0.999	1.385	287.461	207.529	ENSG00000110651	0.470
**ANAPC2**	0.958	1.389	98.922	71.196	ENSG00000176248	0.474
**PXN**	0.996	1.405	447.702	318.605	ENSG00000089159	0.491
**FAM222B**	0.988	1.409	227.494	161.458	ENSG00000173065	0.495
**PUM2**	0.958	1.410	3420.849	2425.903	ENSG00000055917	0.496
**SLC52A1**	0.988	1.414	82.256	58.163	ENSG00000132517	0.500
**CCNJ**	0.990	1.415	262.677	185.679	ENSG00000107443	0.500
**ZNF707**	0.999	1.441	69.418	48.181	ENSG00000181135	0.527
**RBMS3-AS2**	0.997	1.444	67.517	46.765	ENSG00000203506	0.530
**CPPED1**	1.000	1.459	342.650	234.779	ENSG00000103381	0.545
**ABCA2**	0.969	1.461	365.401	250.101	ENSG00000107331	0.547
**ELOVL6**	0.965	1.461	401.088	274.519	ENSG00000170522	0.547
**MLXIP**	0.978	1.463	484.369	331.032	ENSG00000175727	0.549
**RP11-819C21.1**	0.962	1.485	112.200	75.534	ENSG00000261098	0.571
**MAP1S**	0.990	1.487	131.071	88.123	ENSG00000130479	0.573
**PSPN**	0.978	1.523	73.104	47.982	ENSG00000125650	0.607
**LINC01012**	0.996	1.528	71.443	46.756	ENSG00000281706	0.612
**CTD-2574D22.3**	0.967	1.533	100.095	65.304	ENSG00000279789	0.616
**RPS6KA5**	0.993	1.589	1631.753	1027.161	ENSG00000100784	0.668
**RP11-1C8.5**	1.000	1.594	139.362	87.449	ENSG00000261670	0.672
**HSD17B13**	0.991	1.599	39.254	24.547	ENSG00000170509	0.677
**SOX13**	0.970	1.601	134.247	83.855	ENSG00000143842	0.679
**RHBDD3**	0.994	1.601	85.853	53.622	ENSG00000100263	0.679
**RP11-698F20.3**	0.993	1.604	53.128	33.110	ENSG00000258050	0.682

**CTBP1-AS**	1.000	1.608	50.241	31.247	ENSG00000280927	0.685
**RP1-283E3.4**	0.980	1.616	57.750	35.726	ENSG00000227775	0.693
**RP11-680B3.2**	0.951	1.639	42.041	25.653	ENSG00000240521	0.712
**AC009060.1**	0.974	1.641	84.111	51.245	ENSG00000276989	0.715
**ARSA**	1.000	1.646	72.302	43.928	ENSG00000100299	0.719
**RP11-344N10.5**	0.958	1.649	65.329	39.610	ENSG00000272630	0.722
**SMIM5**	0.963	1.651	30.932	18.737	ENSG00000204323	0.723
**RP11-932O9.10**	0.983	1.653	50.385	30.469	ENSG00000269974	0.725
**RP11-732A21.2**	0.999	1.671	45.850	27.436	ENSG00000260008	0.741
**TMEM213**	1.000	1.673	121.997	72.938	ENSG00000214128	0.742
**LINC00895**	0.957	1.683	86.609	51.472	ENSG00000281548	0.751
**RAB27A**	1.000	1.697	1483.336	874.230	ENSG00000069974	0.763
**DENND2A**	0.981	1.699	57.685	33.945	ENSG00000146966	0.765
**CTD-2313F11.1**	1.000	1.701	148.994	87.598	ENSG00000240535	0.766
**RRN3P2**	0.993	1.702	234.453	137.708	ENSG00000103472	0.768
**RP11-769O8.1**	0.999	1.705	53.132	31.152	ENSG00000266171	0.770
**RP11-572C21.1**	0.968	1.719	48.689	28.318	ENSG00000251536	0.782
**SVOP**	0.999	1.727	59.930	34.696	ENSG00000166111	0.788
**LA16c-431H6.7**	1.000	1.747	42.537	24.346	ENSG00000278987	0.805
**CTB-78F1.1**	0.992	1.758	54.164	30.813	ENSG00000254187	0.814
**ENPP6**	0.985	1.768	48.255	27.292	ENSG00000164303	0.822
**AC098820.4**	0.993	1.768	36.949	20.892	ENSG00000241520	0.822
**CXorf36**	0.990	1.783	51.417	28.825	ENSG00000147113	0.835
**C14orf79**	0.985	1.789	24.323	13.593	ENSG00000140104	0.839
**RP11-496B10.3**	0.970	1.799	25.385	14.109	ENSG00000239774	0.847
**FKBP11**	1.000	1.801	602.395	334.439	ENSG00000134285	0.849
**FILIP1L**	0.998	1.802	123.312	68.428	ENSG00000168386	0.850
**STAG3L1**	0.986	1.802	59.073	32.774	ENSG00000205583	0.850
**SRCAP**	0.967	1.805	1092.333	605.014	ENSG00000080603	0.852
**MIDN**	1.000	1.809	152.706	84.427	ENSG00000167470	0.855
**Metazoa**	1.000	1.815	32.199	17.740	ENSG00000276493	0.860
**Metazoa**	0.952	1.816	220.674	121.504	ENSG00000277396	0.861
**SETD1B**	0.970	1.817	274.291	150.914	ENSG00000139718	0.862
**CTD-2231E14.5**	0.995	1.857	17.963	9.671	ENSG00000267373	0.893
**FRMD5**	0.990	1.858	184.466	99.291	ENSG00000171877	0.894
**CTD-2017D11.1**	0.995	1.878	42.970	22.870	ENSG00000268362	0.910
**CLDND2**	0.996	1.896	15.452	8.143	ENSG00000160318	0.923
**RP11-122A21.2**	0.988	1.902	20.402	10.724	ENSG00000250267	0.927
**KCTD4**	0.980	1.911	20.925	10.943	ENSG00000180332	0.935
**RPS2P44**	0.993	1.934	22.790	11.776	ENSG00000243609	0.952
**CTD-3222D19.4**	0.997	1.950	50.576	25.927	ENSG00000279977	0.964
**AL049794.1**	0.975	1.952	36.179	18.526	ENSG00000280240	0.965
**MOV10L1**	0.995	1.976	16.214	8.198	ENSG00000073146	0.983
**LCN15**	0.982	1.998	36.871	18.446	ENSG00000177984	0.999
**TPM3P7**	0.963	2.037	27.545	13.519	ENSG00000187536	1.026
**C19orf68**	0.995	2.037	40.856	20.048	ENSG00000185453	1.027
**AC000120.7**	0.995	2.038	36.393	17.848	ENSG00000243107	1.027
**RP11-133K1.9**	0.981	2.052	25.766	12.552	ENSG00000279409	1.037
**HNRNPMP1**	0.996	2.072	24.211	11.680	ENSG00000259335	1.051
**RP11-184M15.2**	0.983	2.082	12.974	6.227	ENSG00000248802	1.058

**E2F3-IT1**	0.976	2.091	15.965	7.629	ENSG00000224707	1.064
**RP11-756J15.3**	1.000	2.095	28.921	13.797	ENSG00000267177	1.067
**RP11-1017G21.4**	0.982	2.106	14.313	6.792	ENSG00000258959	1.074
**NRBF2P5**	0.994	2.165	10.187	4.699	ENSG00000270427	1.115
**CHERP**	0.996	2.174	173.417	79.758	ENSG00000085872	1.120
**Metazoa**	0.964	2.184	14.111	6.456	ENSG00000274742	1.127
**HLA-G**	0.978	2.204	139.862	63.459	ENSG00000204632	1.140
**PKIB**	1.000	2.247	42.870	19.072	ENSG00000135549	1.168
**RP11-456K23.1**	0.969	2.250	9.830	4.363	ENSG00000267414	1.170
**RP11-46O21.2**	0.995	2.252	28.983	12.865	ENSG00000232342	1.171
**MTND4P26**	0.993	2.252	12.062	5.350	ENSG00000234065	1.171
**RN7SKP271**	0.986	2.271	13.235	5.821	ENSG00000222460	1.184
**BEAN1**	0.982	2.281	18.752	8.215	ENSG00000166546	1.190
**RN7SL491P**	0.987	2.284	11.134	4.868	ENSG00000264400	1.192
**ASPN**	0.980	2.341	58.373	24.929	ENSG00000106819	1.227
**Metazoa**	0.994	2.373	14.476	6.095	ENSG00000275287	1.247
**AC017006.3**	0.961	2.405	11.734	4.873	ENSG00000231336	1.266
**CTC-421K24.1**	0.958	2.414	10.448	4.323	ENSG00000275839	1.271
**RN7SL650P**	0.987	2.429	13.482	5.545	ENSG00000274475	1.280
**AC019048.1**	0.956	2.454	16.756	6.821	ENSG00000223884	1.295
**AC130709.1**	0.999	2.503	12.469	4.976	ENSG00000214525	1.324
**RP11-525G13.2**	1.000	2.516	15.619	6.201	ENSG00000236364	1.331
**RP11-379F4.4**	0.998	2.554	16.685	6.527	ENSG00000240207	1.353
**GRHL2**	0.965	2.588	16.883	6.517	ENSG00000083307	1.372
**OGN**	0.996	2.602	96.461	37.071	ENSG00000106809	1.379
**BRD7P4**	0.994	2.627	13.279	5.049	ENSG00000218676	1.393
**RLIMP1**	0.986	2.654	48.712	18.345	ENSG00000229456	1.408
**KRT18P57**	0.978	2.666	24.725	9.267	ENSG00000215867	1.415
**DPRXP3**	0.997	2.678	14.719	5.490	ENSG00000282308	1.421
**CTC-303L1.2**	0.991	2.685	8.221	3.055	ENSG00000270133	1.425
**SSXP10**	0.959	2.689	8.401	3.118	ENSG00000217330	1.427
**RP11-216N14.7**	0.962	2.702	39.508	14.618	ENSG00000223599	1.434
**RP11-529H22.1**	0.959	2.718	7.616	2.795	ENSG00000250980	1.443
**PHBP19**	1.000	2.727	21.857	8.008	ENSG00000257246	1.447
**Metazoa**	0.999	2.741	7.942	2.891	ENSG00000277039	1.455
**ECM2**	0.998	2.742	117.550	42.865	ENSG00000106823	1.455
**PMS2P2**	1.000	2.773	62.105	22.392	ENSG00000278416	1.471
**AC002306.1**	0.999	2.800	28.790	10.276	ENSG00000259242	1.485
**RPL26P35**	0.993	2.843	11.971	4.204	ENSG00000244229	1.507
**RPL21P8**	1.000	2.866	11.771	4.101	ENSG00000180662	1.519
**RN7SKP130**	0.975	2.870	11.679	4.062	ENSG00000201794	1.521
**RP11-201O14.2**	0.998	2.872	11.050	3.841	ENSG00000232768	1.522
**RP11-463D19.1**	0.972	2.899	19.716	6.796	ENSG00000254538	1.535
**ZNF750**	0.989	2.913	9.835	3.369	ENSG00000141579	1.543
**RP5-837J1.4**	0.997	2.922	24.684	8.442	ENSG00000267457	1.547
**AC114763.1**	0.982	2.951	5.070	1.711	ENSG00000230569	1.561
**ZNF890P**	1.000	3.028	12.974	4.279	ENSG00000159904	1.598
**RP11-116K4.1**	0.967	3.038	4.773	1.565	ENSG00000264296	1.603
**CTD-2647E9.3**	1.000	3.039	69.387	22.822	ENSG00000259188	1.604
**RP11-101O6.2**	0.972	3.104	6.749	2.167	ENSG00000234937	1.634

**OMD**	0.998	3.131	66.408	21.201	ENSG00000127083	1.647
**CTD-2666L21.2**	1.000	3.139	11.389	3.621	ENSG00000234750	1.650
**PIN4P1**	0.999	3.175	11.721	3.685	ENSG00000227973	1.667
**RP11-213H15.4**	0.977	3.522	17.382	4.928	ENSG00000271762	1.817
**RP11-190D6.1**	0.979	3.526	11.483	3.249	ENSG00000279991	1.818
**GAPDHP61**	0.964	3.546	15.161	4.268	ENSG00000248415	1.826
**CCDC116**	0.976	3.582	6.773	1.883	ENSG00000161180	1.841
**LA16c-380F5.3**	0.978	3.607	4.650	1.282	ENSG00000280231	1.851
**PALM2**	0.994	3.621	31.990	8.827	ENSG00000243444	1.856
**RP11-173G21.1**	0.992	3.649	10.158	2.777	ENSG00000230221	1.867
**RPS3P7**	0.957	3.665	5.688	1.545	ENSG00000243101	1.874
**TDGF1P6**	0.969	3.682	11.487	3.113	ENSG00000241438	1.880
**ASPDH**	0.972	3.682	5.114	1.382	ENSG00000204653	1.880
**HLA-DQA2**	0.953	3.782	75.794	20.032	ENSG00000237541	1.919
**RP11-343B5.1**	0.979	3.807	6.417	1.678	ENSG00000233597	1.929
**RP11-328K2.1**	0.968	3.925	11.281	2.866	ENSG00000254048	1.973
**RP11-63P12.7**	0.963	3.928	6.574	1.666	ENSG00000235523	1.974
**RP11-131O15.2**	0.988	3.968	11.591	2.913	ENSG00000228523	1.988
**SETP10**	0.991	4.077	6.893	1.683	ENSG00000231905	2.028
**CTB-174O21.2**	0.966	4.228	4.242	0.996	ENSG00000269487	2.080
**RP11-562A8.1**	0.997	4.278	5.215	1.212	ENSG00000242737	2.097
**SCARNA15**	0.991	4.457	37.705	8.451	ENSG00000280466	2.156
**RP11-567M16.5**	0.999	4.499	5.178	1.143	ENSG00000266901	2.169
**RPL9P30**	0.969	4.579	5.269	1.143	ENSG00000240074	2.195
**XXbac-BPG181M17.6**	0.991	4.592	6.722	1.456	ENSG00000263756	2.199
**BPIFB9P**	0.970	4.664	3.730	0.792	ENSG00000125997	2.221
**RP11-10J21.2**	1.000	4.688	7.561	1.605	ENSG00000280035	2.229
**RPL31P58**	0.998	4.695	4.755	1.005	ENSG00000243053	2.231
**RP11-474I11.8**	0.981	4.695	4.428	0.935	ENSG00000264829	2.231
**CPA5**	0.975	4.719	14.948	3.160	ENSG00000158525	2.238
**LINC00536**	0.992	4.854	24.542	5.048	ENSG00000249917	2.279
**RP11-697N18.1**	0.959	4.994	7.557	1.505	ENSG00000251354	2.320
**RP11-90D4.4**	0.988	5.117	4.825	0.935	ENSG00000277952	2.355
**RN7SKP253**	0.965	5.121	4.594	0.889	ENSG00000252233	2.356
**PNMAL1**	0.998	5.196	16.083	3.087	ENSG00000182013	2.377
**RP11-707O23.1**	0.995	5.277	27.966	5.292	ENSG00000280022	2.400
**IDSP1**	0.978	5.448	2.466	0.444	ENSG00000176289	2.446
**EIF1AXP2**	0.998	5.509	9.150	1.653	ENSG00000258980	2.462
**MIR3180-1**	0.986	5.513	10.694	1.932	ENSG00000258354	2.463
**AC022173.2**	0.966	5.744	3.307	0.568	ENSG00000237243	2.522
**RP11-706O15.5**	0.998	5.753	74.717	12.980	ENSG00000205663	2.524
**RP11-707O23.5**	0.981	5.823	21.749	3.727	ENSG00000263503	2.542
**RP11-138C9.1**	0.995	5.859	36.709	6.258	ENSG00000264558	2.551
**MTRNR2L8**	0.999	5.875	423.203	72.025	ENSG00000255823	2.555
**CTD-2196E14.7**	0.999	5.902	11.406	1.924	ENSG00000279618	2.561
**ABC12-49244600F4.4**	0.978	6.020	4.782	0.786	ENSG00000276144	2.590
**KRT17P8**	0.993	6.181	19.102	3.082	ENSG00000256937	2.628
**Z84812.4**	0.959	6.356	2.163	0.332	ENSG00000260803	2.668
**PI4K2B**	0.994	6.506	26.386	4.047	ENSG00000281028	2.702
**RP11-635L1.2**	0.994	6.628	5.742	0.858	ENSG00000279384	2.728

**NPM1P27**	0.999	6.741	664.538	98.574	ENSG00000249353	2.753
**RP11-806L2.6**	0.979	7.114	5.745	0.799	ENSG00000265490	2.831
**TMEM189-UBE2V1**	0.992	7.260	8.560	1.171	ENSG00000124208	2.860
**RHOXF2**	0.954	7.356	2.243	0.296	ENSG00000131721	2.879
**AC008810.1**	0.984	7.522	5.878	0.773	ENSG00000279126	2.911
**AL135745.1**	0.984	7.522	5.878	0.773	ENSG00000279654	2.911
**CNN3P1**	0.974	8.141	2.207	0.262	ENSG00000218868	3.025
**RP11-759F5.1**	0.996	8.165	5.177	0.625	ENSG00000236327	3.030
**INMT-FAM188B**	0.981	8.411	3.515	0.409	ENSG00000254959	3.072
**RP11-342M21.2**	0.976	8.505	3.601	0.415	ENSG00000259202	3.088
**AC012442.5**	0.993	9.133	2.504	0.265	ENSG00000243389	3.191
**CTD-2192J16.24**	0.983	9.939	4.889	0.483	ENSG00000269590	3.313
**RP11-293B7.1**	0.985	10.961	2.574	0.226	ENSG00000270994	3.454
**SLC25A47**	0.989	11.096	1.745	0.148	ENSG00000140107	3.472
**IGLV5-52**	0.999	11.179	4.482	0.392	ENSG00000211643	3.483
**UTS2**	1.000	11.349	55.628	4.893	ENSG00000049247	3.504
**RP11-49O14.3**	0.966	11.449	2.301	0.192	ENSG00000230815	3.517
**RP11-436M15.1**	1.000	11.825	4.107	0.338	ENSG00000242888	3.564
**MTND6P4**	0.996	12.814	24.114	1.873	ENSG00000249119	3.680
**RN7SL448P**	0.992	13.032	3.573	0.265	ENSG00000264322	3.704
**RNU6-820P**	0.953	13.340	2.658	0.190	ENSG00000207000	3.738
**RPS15AP6**	1.000	13.375	5.113	0.373	ENSG00000233072	3.741
**CLEC4F**	1.000	13.410	27.980	2.077	ENSG00000152672	3.745
**CTD-2193P3.2**	0.993	13.442	3.964	0.286	ENSG00000249772	3.749
**HLA-U**	1.000	13.830	12.769	0.914	ENSG00000228078	3.790
**RP5-1115A15.2**	0.997	14.152	3.401	0.231	ENSG00000270282	3.823
**AC006538.4**	0.985	14.451	3.132	0.207	ENSG00000267001	3.853
**RP4-537K23.4**	0.985	16.837	2.081	0.114	ENSG00000235189	4.074
**ATP6V1B1**	1.000	17.897	4.170	0.224	ENSG00000116039	4.162
**RP11-14C22.6**	0.989	19.949	2.468	0.114	ENSG00000230720	4.318
**RP11-335G20.7**	0.999	21.633	2.770	0.119	ENSG00000259984	4.435
**CTC-297N7.1**	0.995	26.010	2.265	0.077	ENSG00000261433	4.701
**MTATP8P1**	1.000	28.448	21.860	0.759	ENSG00000240409	4.830
**FAM136BP**	0.999	32.977	3.028	0.082	ENSG00000232654	5.043
**AC093249.1**	1.000	34.753	2.938	0.075	ENSG00000280843	5.119
**BCL2L2-PABPN1**	1.000	40.271	4.199	0.095	ENSG00000258643	5.332
**AC004556.1**	1.000	258.291	55.860	0.206	ENSG00000276345	8.013
**DNAJC19P9**	1.000	495.434	4.944	0.000	ENSG00000258608	8.953
**TBC1D3G**	1.000	1190.662	11.897	0.000	ENSG00000260287	10.218
